# Imaging mass cytometry unveils functional and spatial remodeling of peri-lesional cells in jaw osteonecrosis

**DOI:** 10.1038/s42003-026-09696-7

**Published:** 2026-02-16

**Authors:** Jiazheng Cai, Ying Xue, Stian Tornaas, Harsh Nitin Dongre, Athanasia Bletsa, Sigbjørn Løes, Peter Schleier, Evelyn Neppelberg, Arild Kvalheim, Ellen Berggreen, Daniela-Elena Costea, Zhe Xing, Anca Virtej

**Affiliations:** 1 Department of Medical Biology, Faculty of Health Sciences, UiT the Arctic University of Norway, Tromsø, Norway; 2 Department of Clinical Dentistry, Faculty of Health Sciences, UiT the Arctic University of Norway, Tromsø, Norway; 3 Gade Laboratory for Pathology and Norwegian Center for Cancer Biomarkers CCBIO, Department of Clinical Medicine, Faculty of Medicine, University of Bergen, Bergen, Norway; 4 Department of Clinical Dentistry, University of Bergen, Bergen, Norway; 5 Department of Oral and Maxillofacial Surgery, Haukeland University Hospital, Bergen, Norway; 6 Department of Otolaryngology, Stavanger University Hospital, Stavanger, Norway; 7 Oris Dental Tannteam Secialist Clinic, Nesttun, Norway; 8 Department of Biomedicine, University of Bergen, Bergen, Norway; 9 Department of Pathology, Haukeland University Hospital, Bergen, Norway

**Keywords:** Pathogenesis, Oral diseases

## Abstract

Medication-related osteonecrosis of the jaw (MRONJ) is a severe complication associated with antiresorptive therapy, characterized by compromised bone and soft tissue integrity. However, the underlying tissue-level mechanisms remain poorly understood. To uncover cell functions and spatial organization surrounding ONJ lesions, imaging mass cytometry is used to profile lesions at single-cell resolution across epithelial, stromal, and vascular regions. Widespread immune infiltration is observed, with regulatory T cells, M2-like macrophages, exhausted T cells, and natural killer cells shifting from dispersed to clustered spatial patterns, indicating altered immune organization. Epithelial regions show reduced epithelial marker expression and disrupted architecture despite elevated proliferation-related markers, while fibroblasts and endothelial cells display signs of activation. Functional profiling reveals concurrent proliferative, apoptotic, and stress-associated signatures across multiple cell populations. This comprehensive spatial and functional atlas provides insights into the pathophysiology of MRONJ and may inform future therapeutic strategies aimed at restoring tissue homeostasis and promoting effective healing.

## Abstract (web summary)

Imaging mass cytometry maps the peri-lesional microenvironment in medication-related osteonecrosis of the jaw, revealing altered immune spatial organization, impaired epithelial regeneration, and differed functional profiling

## Introduction

Medication-related osteonecrosis of the jaw (MRONJ) is a chronic, progressive disorder characterized by necrosis of the jawbone and persistent exposure of oral mucosa. The primary trigger of MRONJ is the administration of antiresorptive agents, such as bisphosphonates and denosumab, which are widely used in the treatment of osteoporosis, metastatic bone diseases, and multiple myeloma^1^. Although these medications are effective in suppressing bone resorption, a subset of patients develop MRONJ, typically in the presence of local inflammation or infection, particularly following dental procedures.

The pathophysiology of this disease is complex and controversial. Current theories emphasize several interrelated mechanisms, including suppression of bone remodeling, local infection, inhibition of angiogenesis, immune dysregulation, and possible genetic predispositions^2,3^. Central to all these hypotheses is the role of the local immune microenvironment and its interaction with bone and mucosal tissues. Various types of cells, such as immune cells, fibroblast, endothelial cells, epithelial cells and bone cells, are implicated in the disease process^4–6^. However, the precise immune cell behavior and spatial distribution of each in the lesional tissue remain poorly understood. In particular, the functional states of these cells, such as proliferation, activation, stress-response and initiation of programmed cell death in distinct tissue regions are not well characterized. A deeper understanding of these interactions at the single-cell level is essential for elucidating MRONJ etiology and identifying therapeutic targets.

Spatial proteomics provides a multidimensional view of protein expression and localization within intact tissue architecture, enabling the study of cellular phenotypes, interactions, and microenvironmental organization in human disease^7^. Among different spatial proteomic technologies, Imaging Mass Cytometry (IMC) offers a practical and robust solution for high-plex proteomic profiling of Paraffin-Embedded (FFPE) tissues^8,9^. By using stable metal isotopes, IMC avoids the spectral overlap limitations of traditional immunofluorescence and permits simultaneous detection and imaging of over 40 markers on a single tissue section^10^. Compared with multiplexed ion beam imaging (MIBI), which also relies on metal-tagged antibodies, IMC benefits from broader commercial antibody panel availability, streamlined sample preparation, and wider deployment in clinical and research pathology settings^11,12^.

Tissue used in IMC is ablated using a high-resolution 1 μm laser, and the released metal tags are quantified via time-of-flight mass spectrometry, enabling spatially resolved proteomic profiling^13^. This approach provides clear insights into the phenotypic diversity and spatial organization of cells in complex tissues. Downstream analysis platforms such as QuPath (released under GNU GPLv3 license), an open-source image analysis tool, facilitate marker quantification and cell segmentation^14^. SpicyR, an R package tailored for spatial proteomics, enables statistical analysis of spatial relationships and cellular neighborhoods^15^. Together, these tools offer a powerful framework to dissect the cellular microenvironment of MRONJ and explore the intercellular interactions driving disease progression.

In this study, IMC approach was employed to systematically characterize the cellular composition, functional states, and spatial architecture of ONJ peri-lesional tissues at single-cell resolution. By integrating IMC with complementary immunohistochemistry (IHC) validation, this work aims to provide a spatial atlas of the viable peri-lesional microenvironment. The findings are expected to deepen the understanding of epithelial dynamics, immune cell infiltration, and tissue remodeling in ONJ, ultimately contributing to the identification of potential therapeutic targets and novel strategies for restoring local tissue homeostasis.

## Results

### Region-specific changes in cell composition and functional states in ONJ

**Fig. 1:**
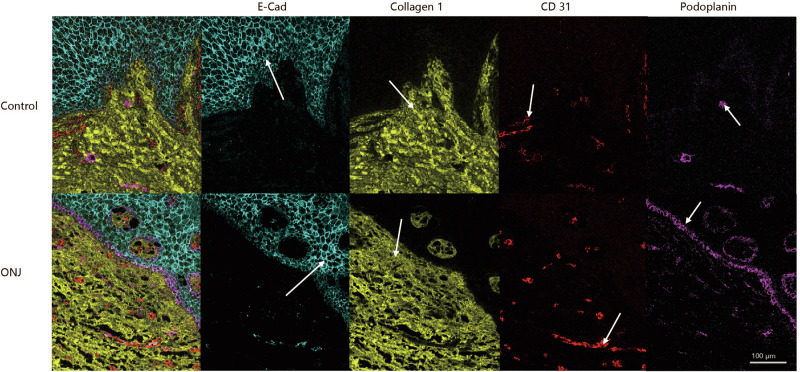
Visualization of epithelial, stromal, and vascular structures through MCD. E-cadherin (cyan) staining the epithelia, collagen-1 (yellow) staining the stroma and CD31 (red) and podoplanin (magenta) staining the blood vessels and lymph vessels, respectively. White arrows for positive staining. Scale bar: 100 μm.

Based on morphological features and marker expression, tissue sections, three major anatomical regions: epithelium, stroma, and vessels were identified in both control and ONJ tissue samples, as shown in Fig. 1. As illustrated in Supplementary Fig. 1a, QuPath-based annotations revealed an interdigitated junction between the basal layer of the epithelium and the underlying connective tissue, while vessel regions displayed basically typical vascular morphologies. Vessel regions were primarily located within stromal areas, and some were also observed within the epithelial compartment.

**Fig. 2:**
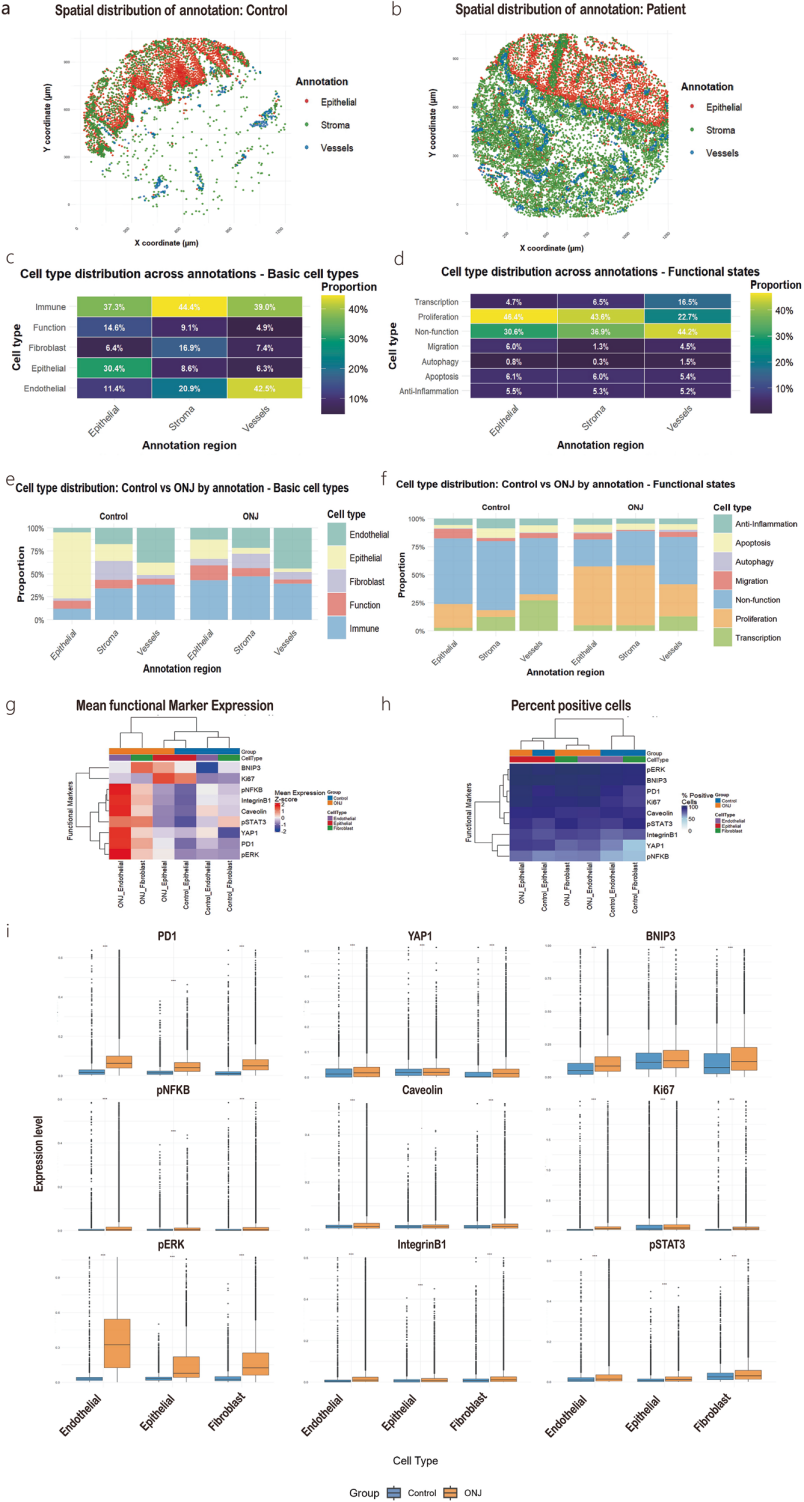
Regional annotation and cellular composition in Control and ONJ tissues. Representative regional segmentation of single tissue sections from Control (**a**) and ONJ (**b**) groups, showing epithelium, stroma, and vessel regions. Heatmaps showing the distribution of major cell types (**c**) and functional cell types (**d**) across annotated regions. **e**, **f** Comparison of cell type proportions between Control and ONJ groups. **g** Heatmap of functional marker expression levels across different regions in both groups. **h** Percentage of functional marker-positive cells in each region, comparing Control and ONJ samples. (**i**) Expression of functional markers in three basic cell types across Control and ONJ groups. Plots show the median and the interquartile range (box, 25th–75th percentiles). Statistical details of e and f are provided in Supplementary Table 1, statistical details of i are provided in Supplementary Table 2. *n* = 8 Control and *n* = 6 ONJ biologically independent individuals. Significance thresholds: ***p < 0.001; *p < 0.05; ns. *p* > 0.05.

The distribution of single cells across regions is shown in Fig. 2a, 2b, with spatial annotations aligned with those defined in QuPath. Different regions mainly express epithelial cells, fibroblast cells and endothelial cells separately. Immune cells were observed across all tissue regions, including epithelial, stromal, and vessel regions (Fig. 2c). However, due to resolution limitations, the precise localization of immune cells within vascular regions could not be determined. Proliferating cells were mainly enriched in epithelial and stromal regions, while vessel regions were more populated by non-functional or unclassified cells (Fig. 2d). Compared to the control group, a higher overall cell density was observed in ONJ mucosal tissue, consistent with an elevated number of proliferating cells in ONJ patients (*p* < 0.001, Fig. 2f and Supplementary Table 1). Additionally, some fibroblast cells were found within the epithelial compartment in ONJ samples (*p* < 0.001, Fig. 2e, Supplementary Table 1).

Functional characteristics of tissue regions also differed between the groups. In the control group, epithelial regions exhibited stronger higher Ki67 expression, whereas ONJ tissues showed increased expression of multiple markers associated with stress (BNIP3, Caveolin), immune/inflammatory (PD1, pNFκB, pSTAT3), and signaling/migration (IntegrinB1, pERK), (*p *< 0.001; Fig. 2g, i and Supplementary Table 2). Notably, stromal regions in ONJ samples contained a higher proportion of pNFκB-positive cells (Fig. 2h), and this increase was also evident within fibroblasts and endothelial cells (*p* < 0.001; Fig. 2i; Supplementary Table 2), suggesting a potential link to sustained wound-healing activity and vascular dysfunction.

### Cells clustering and spatial relationships

To investigate cellular clustering and spatial relationships, three groups of samples were analyzed: control oral mucosa, ONJ lesions, and tonsil tissue as an immune-rich reference.

**Fig. 3:**
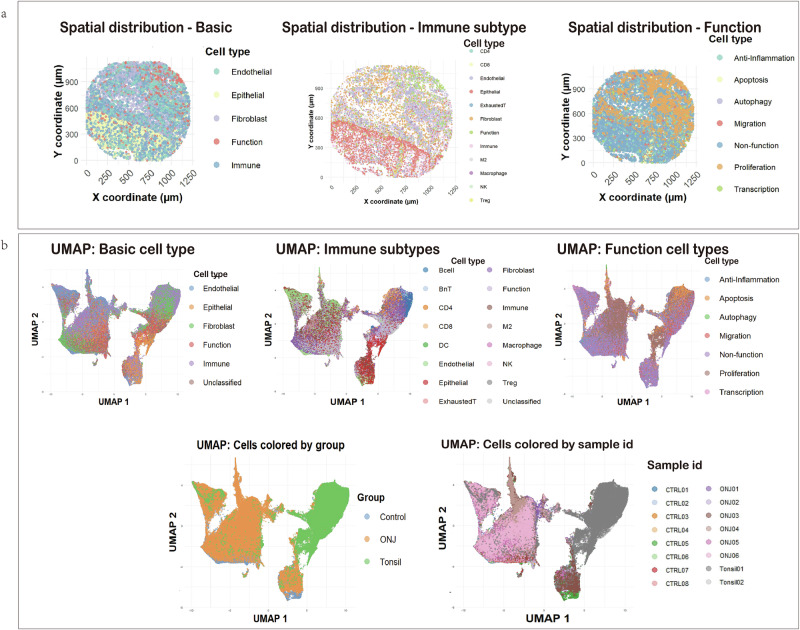
Spatial distribution of cell types and dimensionality reduction analysis. **a** Representative images showing ONJ tissue sections annotated by basic cell types, immune subtypes, and functional states. **b** UMAP plots of all samples combined, colored by cell type, experimental group (Control, ONJ, Tonsil), and sample ID, respectively. Additional UMAPs by individual sample ID and selected marker expression are shown in Supplementary Fig. 4.

The results of single-cell annotation are provided in Supplementary Table 3, with marker validation shown in Supplementary Fig. 2. A representative tissue (Fig. 3a) was used to illustrate spatial distribution and clustering behavior of annotated cell types.

In the UMAP results, tonsil samples formed a distinct cluster, well separated from patient-derived oral tissues. In contrast, control and ONJ samples largely overlapped with no clear separation, suggesting a shared baseline immune landscape (Fig. 3b).

The UMAP of basic cell types showed no preferential cell proximity for immune cells, while among the immune subtypes, B cells, CD4⁺, and CD8⁺ T cells were located in adjacent clusters, although BnT (B cell near T cell) cells appeared to spatially separate the B and T cell populations. Macrophages and M2-like cells exhibited strong spatial adjacency. In the UMAP of functional states, a spatial gradient was observed, ranging from proliferating cells (Ki67, pERK), through apoptotic cells (BNIP3), to autophagic cells (Caveolin), reflecting potential transitions from active cell cycling toward stress responses and eventual degradation within the tissue.

**Fig. 4:**
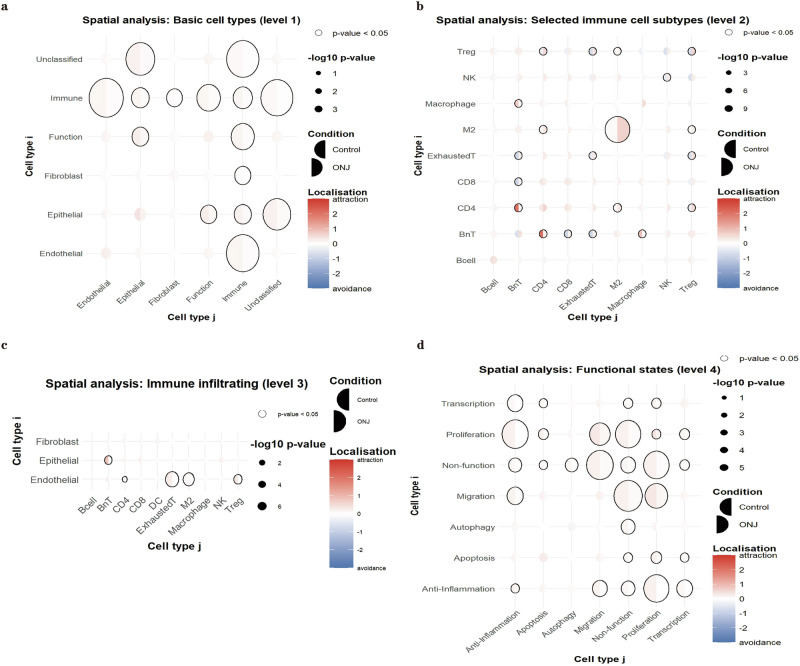
Spatial interaction analysis using the SpicyR package. **a** Spatial co-localization patterns among basic cell types. **b** Spatial interactions among immune subtypes (note: blank for DC due to low cell counts). **c** Spatial relationships between immune subtypes and basic cell types. **d** Spatial proximity among functional cell states. In each plot, the left half-circle represents the Control group, and the right half-circle represents the ONJ group. Larger circles indicate greater differences in spatial interaction strength. Bold black outlines around circles denote statistically significant differences (*p* < 0.05) between groups. Detailed values and statistical outputs are provided in Supplementary Table 7, *n* = 8 Control and *n* = 6 ONJ biologically independent individuals.

Spatial attraction and avoidance between different cell types were also assessed (Fig. 4). In Fig. 4a, c, stronger attraction toward immune cells was observed across basic cell types in control samples, whereas this attraction was significantly reduced in ONJ samples. This pattern suggests that immune cells in ONJ tissues are more spatially dispersed overall.

Further analysis of immune cell subtypes (Fig. 4b) revealed several notable shifts in spatial organization. In control tissues, CD4⁺ T cells and BnT cells exhibited pronounced spatial proximity, which was lost in ONJ tissues, showing no clear tendency toward attraction or avoidance. Similarly, macrophages and BnT cells shifted from clustered in controls to a more neutral pattern in ONJ.

In contrast, certain immune subtypes exhibited increased spatial clustering in ONJ samples. Specifically, exhausted T cells, M2 macrophages, NK cells, and regulatory T cells (Tregs), which showed self-avoidance in control samples, displayed self-attraction in ONJ tissues, indicating localized accumulation. Moreover, several immune subtype pairs reversed their spatial relationships. Tregs and exhausted T cells, BnT and exhausted T cells, BnT and CD8⁺ T cells, and CD4⁺ T cells and Tregs all shifted from avoidance in control tissues to attraction in ONJ, reflecting altered immune cell organization under disease conditions (Fig. 4b).

In addition, functional cell states in ONJ tissues exhibited more dispersed spatial relationships compared to those in control tissues (Fig. 4d), further indicating a breakdown in the structured cellular microenvironment.

### Epithelial cell deficiency and functional alterations in ONJ

**Fig. 5:**
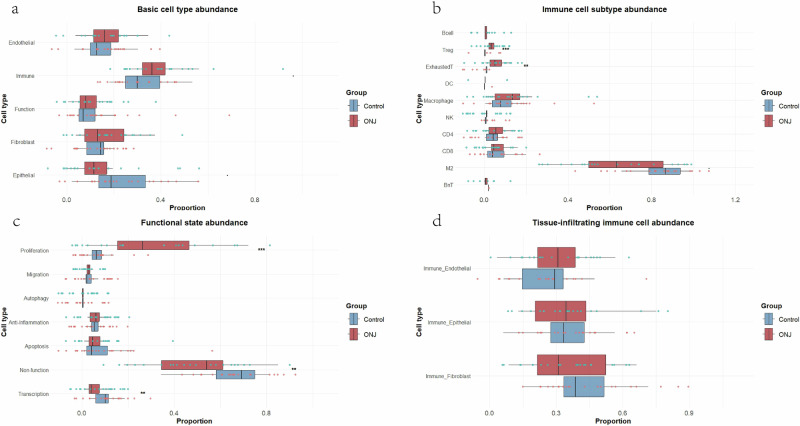
Differential cell abundance between Control and ONJ samples. **a** Basic cell types. **b** Immune cell subtypes. **c** Distribution of functional cell states. **d** Tissue-infiltrating immune cells. B cells were rarely detected in Control samples, resulting in insufficient data for statistical comparison. Detailed values and statistical outputs are provided in Supplementary Table 8. The center line denotes the median, the box spans the interquartile range (25th–75th percentiles), *n *= 8 Control and *n* = 6 ONJ biologically independent individuals. Statistical significance: ***p < 0.001; **p < 0.01; ns. *p* > 0.05.

Epithelial cells were defined based on the expression of E-Cadherin (E-cad) and epidermal growth factor receptor (EGFR), and were found to express high levels of Ki67 and YAP1 (Supplementary Fig. 2). A reduction in epithelial cell abundance was observed in ONJ samples compared to controls (Fig. 5a), with a statistically significant decrease specifically within the annotated epithelial regions (*p* < 0.001; Fig. 2e and Supplementary Table 1).

Epithelial regions in ONJ tissues showed increased YAP1 expression (Fig. 2g), this upregulation was also confirmed at the level of annotated epithelial cells (*p* < 0.001; Fig. 2i). Given the established role of YAP1 in epithelial proliferation, repair, and stemness^16^, this finding suggests that ONJ epithelial cells may be engaged in an altered regenerative program. Rather than reflecting only transitional or misclassified cells, the data supports a broader shift in epithelial cell states within ONJ tissues, potentially linked to stress responses or dysregulated regeneration.

Functional changes in epithelial cells were also evident in ONJ tissues. Several markers, including the apoptosis stress-induced cell death marker BNIP3, the pro-inflammatory marker pNFκB, the proliferation-associated markers Ki67 and pERK, and structural or signaling-related markers such as Caveolin, IntegrinB1, and pSTAT3, were all upregulated in ONJ soft tissues (Fig. 2i, *p* < 0.001). The ONJ group showed significantly increased proportions of proliferating, apoptotic, and autophagic cells compared to controls (*p* < 0.001; Fig. 2f and Supplementary Table 1), suggesting widespread activation of stress and repair-related pathways in the epithelial compartment.

### Increased proliferation and stress marker expression in ONJ vascular regions

The vascular regions were primarily composed of endothelial cells, as shown in Fig. 2c, including both lymphatic endothelial cells (Podoplanin-positive) and blood vascular endothelial cells (positive for CD31, CD140b, and CD146). A slightly higher proportion of endothelial cells was observed in ONJ samples compared to controls, although the difference was not statistically significant (Fig. 5a).

A large fraction of cells within the vascular regions were classified as non-functional (44.2%; Fig. 2d). However, compared to control tissues, ONJ samples exhibited an increased presence of proliferating cells in the vessel regions (Fig. 2f). This was accompanied by elevated expression of multiple functional markers, including Ki67, BNIP3, pERK, pSTAT3, PD1, pNFκB, IntegrinB1, and Caveolin (Fig. 2g). Endothelial cells also showed a significant increase in function markers expression in ONJ compared to control tissues (*p* < 0.001; Fig. 2i).

### Immune cells infiltration and altered spatial preferences

All stromal, vascular, and epithelial regions contained substantial numbers of immune cells (Figs. 2 and 3), indicating widespread immune infiltration across tissue compartments. In ONJ tissues, a marked increase in the number of infiltrating B cells, Tregs, and exhausted T cells was observed compared to controls (Fig. 5b). Notably, the number of B cells in control samples was too low to permit statistical analysis, further highlighting the pronounced accumulation of B cells in ONJ tissues.

Cells in ONJ group also exhibited high expression of PD1(Fig. 2g, 2h), suggesting the presence of immunosuppressive signaling and potential immune escape. Meanwhile, the concurrent upregulation of proliferation markers in these cells may reflect a dynamic balance between immune regulation and tissue repair.

As shown in Supplementary Fig. 2, heatmap of Level 3 basic-immune classification indicates true infiltrating phenotypes rather than segmentation artifacts. Compared to controls, ONJ tissues showed a slightly increased immune cell infiltrating endothelial compartment, though without statistic difference (Fig. 5d).

Spatial interaction analysis revealed distinct shifts in immune-tissue associations. In control samples, BnT cells exhibited stronger spatial attraction to epithelial cells, while CD4⁺ T cells, exhausted T cells, M2 macrophages, and Tregs were more strongly attracted to endothelial regions. Macrophages showed greater spatial association with fibroblast-rich areas in control samples compared to ONJ. These observations suggest a reorganization of immune-tissue interactions in ONJ, potentially reflecting changes in local immune surveillance, immune suppression, or repair mechanisms.

**Fig. 6:**
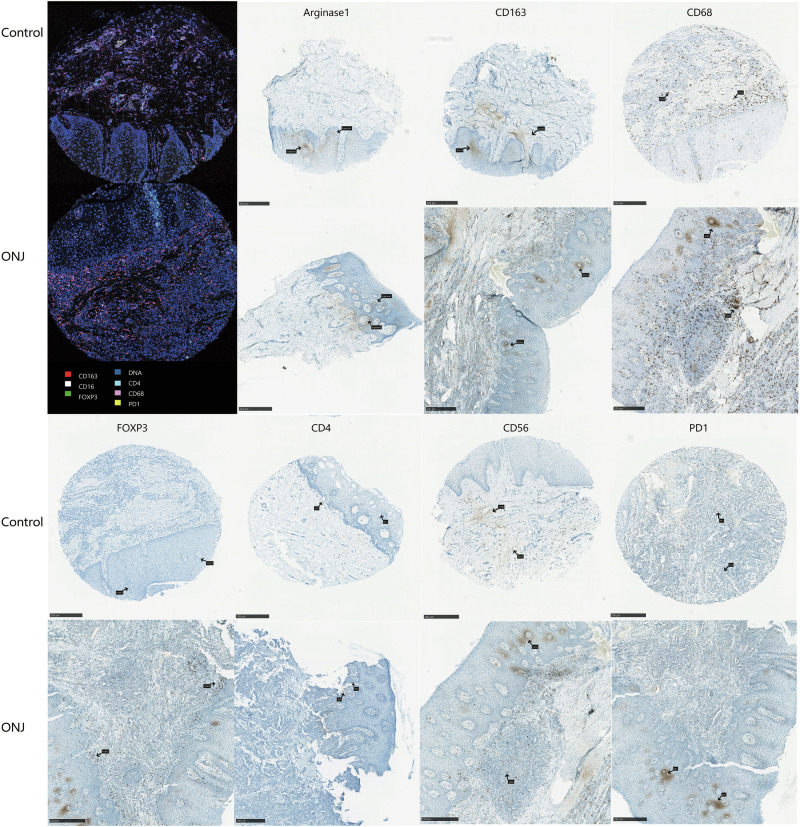
IHC validation of immune cell infiltration patterns. Representative staining images comparing IMC and IHC results for marker expression on CD68^+^ macrophages, Arginase1^+^/ CD163^+^ M2 macrophages, FoxP3^+^ regulatory T cells, CD4⁺ T cells, CD8⁺ T cells, CD20^+^/CD56^+^ B cells, and PD1^+^ exhausted T cells. Black arrows indicate positively stained cells. Scale bar: 250 μm.

To validate the spatial and compositional changes observed by IMC, IHC staining was performed for selected immune markers (Fig. 6). The results confirmed key trends identified in the IMC analysis. M2 macrophages, marked by CD163 and Arginase1, were observed clustering around vessel-like structures in ONJ tissues. CD68^+^ macrophages were also more abundant in ONJ samples. Notably, CD68⁺ macrophages appeared not only in stromal regions, as seen in controls, but also within the epithelium, indicating more superficial infiltration.

CD4⁺ T cells were mainly distributed around vascular regions in both groups, while FOXP3⁺ Tregs were rare and dispersed in controls but appeared more aggregated in ONJ tissues. Similarly, PD1⁺ cells showed minimal presence and scattered distribution in controls, yet were more numerous and locally clustered in ONJ, often located near vessel-like structures. CD56⁺ NK cells were sparse and restricted to stromal regions in controls but were more abundant and broadly distributed across all regions in ONJ.

## Discussion

MRONJ represents a complex pathological condition in which bone and soft tissue integrity is compromised, often in the context of antiresorptive therapy^17,18^. Despite increasing clinical awareness, the underlying mechanisms remain partly understood. In this study, we applied high-dimensional IMC to systematically characterize the tissue microenvironment of ONJ soft tissue, revealing multi-layered changes in cellular composition, functional states, and spatial organization.

Our findings revealed extensive infiltration and redistribution of immune cells across all tissue regions in ONJ samples, including the stroma, epithelium, and vascular compartments (Figs. 2, 3 and 6). The widespread presence of immune cells indicates a persistent inflammatory microenvironment, which may contribute to chronic non-healing lesions. A key observation was the significantly increased expression of PD1 among immune cells in ONJ tissues (Fig. 2g, h and 6). PD1 is a well-known marker of T cell exhaustion and immune suppression^19^. This finding indicates that, although immune cells infiltrate ONJ lesions, their ability to clear damaged cells and support proper healing may be functionally impaired. Furthermore, specific immune subtypes in ONJ tissues exhibited slightly upregulated proliferation markers (Ki67), including B cells, BnT cells, and exhausted T cells (Supplementary Fig. 2), potentially reflecting an ongoing limited proliferative activation in response to local tissue damage or chronic inflammation. The expression of CD4⁺ and CD8⁺ T cells showed no significant increase in ONJ patients (Fig. 5b), which algins with other research results^20^.

Spatial analysis (SpicyR) revealed marked changes in immune cell spatial organization in ONJ tissues compared to controls (Fig. 4). In ONJ samples, Tregs, exhausted T cells, and M2-like macrophages shifted from self-avoidance to self-clustering, suggesting local immunosuppressive niches^21^. These altered microenvironments may limit effective antimicrobial and tissue repair responses by limiting access to the injury site, reducing phagocytic capacity or altering Treg migration and homing^22,23^. Moreover, in ONJ, the attraction between BnT cells and epithelial cells observed in controls was largely lost, and the spatial proximity between CD4⁺ T cells, exhausted T cells, and endothelial regions was also reduced. These changes imply a disruption of coordinated immune surveillance and tissue-specific immune support functions.

IHC analysis further confirmed these spatial and compositional shifts (Fig. 6). ONJ tissues exhibited higher densities and aggregation of Arginase1⁺ M2 macrophages, CD163⁺ macrophages, and FOXP3⁺ Tregs, supporting the presence of a local immunosuppressive microenvironment^24,25^. The increased presence of CD4⁺ T cells, CD56⁺ NK cells, and CD68⁺ macrophages, distributed across epithelial, stromal, and vascular regions, indicates a broader dysregulation of immune cell recruitment and activation rather than a uniform suppressive state. The marked increase in PD1⁺ cells further underscore potential immune exhaustion and impaired effector functions in ONJ tissues^21,26^.

Beyond immune cells, elevated PD-1 expressions were also observed in both epithelial cells and epithelial regions in ONJ tissues (Fig. 2g, i). While PD-1 is classically associated with immune cells, it has also been reported in epithelial cells under conditions of cancer or chronic inflammation^21^. Its upregulation in epithelial compartments may reflect an attempt to modulate local immune responses during chronic tissue injury.

Consistent with the structural damage characteristic of ONJ^1^, our IMC analysis revealed a depletion of epithelial marker (EGFR, E-Cad) expression within epithelial regions of ONJ samples (Fig. 2e). This loss is aligned with our previous findings showing a reduced epidermal growth factor (EGF) expression in areas affected by delayed healing and epithelialization^27^. Interestingly, despite this loss, the epithelial compartment showed an increased proportion of Ki67-positive cells (Fig. 2i), suggesting these cells retain proliferative potential. This contradiction points to a disorganized or insufficient regenerative response, where individual cells may attempt to proliferate but fail to coordinate tissue-level repair^28^.

YAP1 is a well-established regulator of epithelial proliferation, repair, and stemness^16^. In this study, both regional analysis and single-cell measurements demonstrated increased YAP1 expression in ONJ epithelial compartments (Fig. 2). However, given the concomitant structural disorganization and incomplete epithelial reconstitution, the YAP1 activation is more likely to reflect dysregulated or insufficient repair. Other signaling pathways, such as β-catenin, Notch, or TAZ^29–31^ may also influence epithelial behavior in ONJ. Further investigation is needed to clarify whether ONJ epithelium is attempting regeneration or exhibiting broader signaling dysfunction.

Additionally, the expansion of stromal regions into epithelial areas may reflect aberrant wound-healing responses, often driven by fibroblast activation and matrix remodeling^32^. Such maladaptive crosstalk between epithelial and stromal compartments likely exacerbates structural disorganization and perpetuates a chronic non-healing state. Moreover, stromal architecture differed between groups. Control tissues displayed sparsely distributed cells with collagen-rich extracellular matrix, whereas ONJ samples showed substantially increased cellular density and reduced collagen occupancy (Fig. 2a, b and Supplementary Fig. 3). Such differences may reflect pathological stromal changes driven by chronic injury, inflammation, and attempted repair responses. At the same time, variation in stromal composition may also be influenced by differences in tissue origin, as control specimens were obtained from soft tissues surrounding third-molar extractions, whereas ONJ samples were collected from gingival, palatal, or buccal mucosa adjacent to lesions (Supplementary Table 4).

Finally, vascular regions in ONJ tissues also showed slight changes. Although the increase in endothelial cells in ONJ tissues was not statistically significant (Fig. 5a), ONJ samples exhibited visibly expanded vascular areas (Fig. 2b). The enlarged vascular compartments may be consistent with inflammation-associated vascular dilation^33^. This interpretation is supported by elevated pNFκB expression within vascular regions of ONJ samples (Fig. 2g), indicating inflammatory activation, together with significantly increased BNIP3 levels in endothelial cells (Fig. 2i), suggesting hypoxic stress and compromised endothelial integrity^34^. These findings imply that although vascular spaces appear expanded, the functional state of endothelial cells may be impaired. Meanwhile, ONJ tissues demonstrated increased expression of pERK, PD1, YAP1, Caveolin, and IntegrinB1 across epithelial, stromal, and vascular regions (Fig. 2g). Rather than reflecting enhanced function within any specific compartment, these changes likely represent a broader stress-related activation pattern associated with chronic inflammation and dysregulated tissue repair^31,35–37^.

In addition to changes in immune infiltration and structural cell composition, ONJ tissues displayed marked heterogeneity in cellular functional states. Our IMC analysis revealed an overall significant increase in proliferative cells and a marked decrease in transcriptionally active cells in ONJ tissues compared to controls (Fig. 5c). Region-specific analysis (Fig. 2f) further confirmed these changes in different compartments.

ONJ tissues displayed a striking imbalance between proliferative and stress-related responses. Ki67 expression was elevated, indicating persistent proliferative activity, but this was paralleled by increased BNIP3 levels (Fig. 2), a marker of apoptosis and hypoxia-induced stress^38^. The coexistence of these signals suggests that regenerative attempts occur in the context of ongoing cellular damage and metabolic dysregulation. Moreover, increased expression of signaling markers such as pERK, pSTAT3, Caveolin, and IntegrinB1 in multiple cell types (Fig. 2i) indicates an attempt to activate compensatory pathways for survival, proliferation, and migration under stressful conditions^35–37,39^.

These observations reveal a complex functional imbalance in ONJ tissues, where simultaneous activation of proliferative, apoptotic, and stress-related pathways coexists without successful resolution. This functional heterogeneity likely contributes to the chronic non-healing nature of ONJ lesions, highlighting the importance of targeting both regenerative and stress pathways in future therapeutic strategies.

While this study provides a comprehensive spatial and functional characterization of ONJ tissues, certain methodological aspects warrant consideration. Patients with ONJ do not always undergo surgical treatment, resulting in a paucity of samples that were available for our analysis. Furthermore, due to necrotic areas, several samples from patients with ONJ had to be excluded from further analysis. An increased number of samples would have provided more insights into the pathophysiology of tissue affected by ONJ. Due to ethical restrictions which secured patient confidentiality, the detailed clinical information, including patients’ medical history and medication regimens, was not available. As such, clinical contexts were not correlated with our analyses. The absence of detailed clinical metadata may introduce uncertainty, as these factors can influence tissue architecture, inflammation, and immune phenotypes. Without such covariates, it is difficult to fully understand biological variation from potential differences driven by clinical background or treatment history. Future studies will seek ethical approval to access more de-identified clinical information and to include a larger number of patient samples, with the goal of distinguishing molecular and spatial features across different stages of the disease.

In this study, tissue cores were obtained from multiple ONJ and control samples with diverse anatomical origins and histological characteristics (Supplementary Table 4). Necrotic regions were not included in the analysis due to both technical and biological constraints. During FFPE processing, necrotic soft tissue is frequently lost because it becomes fragmented or detaches during dehydration and clearing. Even when preserved, such regions display severely degraded nuclear morphology and markedly reduced antigenicity, precluding reliable cell segmentation or marker quantification. From a biological perspective, necrotic zones contain few intact cells and therefore provide limited information regarding signaling states or spatial interactions within the tissue microenvironment.

UMAP-based dimensionality reduction revealed no clear group-level separation between ONJ and control samples, suggesting partially shared cellular states across disease and non-disease tissues. In contrast, tonsil-derived samples formed a distinct cluster, consistent with their lymphoid composition and immune-enriched microenvironment (Supplementary Fig. 4). Within both ONJ and control groups, inter-sample heterogeneity was observed, likely attributable to differences between patients within epithelial structure, connective tissue composition, and local vascular features. All control tissues were collected from the vestibular fold near the wisdom tooth, yet varied in epithelial keratinization and stromal density.

For example, Control 1 and Control 2 both featured parakeratinized epithelium and fibrous connective tissue and displayed similar UMAP distributions. However, Control 2, noted for more dilated vessels, showed increased representation within CD146⁺ endothelial clusters, suggesting that subtle histological differences such as vascular dilation may influence local cell phenotypes. In contrast, Control 3 and Control 6, which exhibited looser connective tissue and non- or partially keratinized epithelium, showed more localized clustering patterns, potentially reflecting reduced cellular complexity and distinct tissue architecture.

Among ONJ samples, P1, P2, and P3 were derived from similar mucosal regions but exhibited variation in cell-type composition and clustering, which may reflect inter-patient differences in inflammation status or antiresorptive treatment response. P5 and P6, sourced from gingival defect regions, showed enriched expression of stromal and immune markers. Both samples exhibited higher levels of CD68, collagen-1, CD90, and αSMA, indicating increased macrophage infiltration, fibroblast activity, and myofibroblast differentiation, respectively (Supplementary Fig. 4). Notably, P5 showed elevated CD163 expression, suggesting a predominance of M2-like macrophages, whereas P6 displayed enhanced pERK signaling, which may reflect active tissue remodeling or stress-induced signaling pathways. These distinct profiles suggest that local microenvironmental stimuli, such as mechanical forces or ongoing repair processes, may drive differential cellular responses even within gingival ONJ lesions.

In the design of the IMC antibody panel, key immune markers such as CD3 and CD45 were not included. Although this limitation may reduce the resolution of certain immune subpopulations, we mitigated this by optimizing marker combinations and hierarchically adjusting the marker ranking strategy. Furthermore, validation using IHC staining for major immune markers was performed to strengthen the interpretation of immune cell subsets.

Regarding data analysis, a hybrid workflow combining QuPath and R was utilized. This approach provides intuitive visualization and user-friendly operation for biologists, while retaining the advantages of reproducible, high-throughput quantification afforded by code-based pipelines. For dimensionality reduction, UMAP parameters were carefully chosen to balance local and global data structure, enhancing interpretability of cellular heterogeneity and spatial organization. The specific settings used in this study allowed robust identification of cell clusters and neighborhood relationships, supporting reliable downstream biological interpretations.

Overall, these methodological considerations reflect the balance between technical feasibility and biological insight in this study. Future work integrating more detailed clinical metadata, expanded marker panels, and complementary analytical frameworks may further deepen our understanding of ONJ microenvironment dynamics.

## Materials and methods

### Tissue collection and preparation

Human oral mucosa and gingival tissue samples from MRONJ lesions and healthy controls, as well as healthy tonsils were collected at the Department of Maxillofacial and Oral Surgery, Haukeland University Hospital, Bergen, Stavanger University Hospital, Stavanger, Norway, and Tannteam Private Dental Clinic, Bergen, Norway. Informed consent was obtained from all participants. The study was approved by the Regional Committees for Medical and Health Research Ethics (REK), approval number 2018/716. All ethical regulations relevant to human research participants were followed.

ONJ samples from MRONJ patients were obtained during surgical debridement procedures. Control samples were collected during routine oral surgeries, such as impacted third-molar extractions. Tonsil specimens obtained from elective tonsillectomy served as positive controls for the IMC and IHC procedure. All tissues were formalin-fixed, frozen and stored at -80° until further analysis. Samples were then paraffin-embedded before histological and IMC analysis. Core sections from each sample were stained with Hematoxylin and Eosin (HE) for visualization of tissue structure. Samples with necrotic tissue were excluded from further analyses. The core tissue micro-array of samples subjected to IMC analysis is shown in supplementary Fig. 5.

For IHC analysis, sections from selected FFPE blocks were stained for Arginase1, CD163, CD68, FOXP3, CD4, CD56, and PD1 to validate immune cell composition and spatial distribution identified in the IMC analysis. Tissue sections (3–5 μm) were deparaffinized, rehydrated, and subjected to antigen retrieval in pH 6 citrate buffer using a pressure cooker (BioCare Medical, Model DC2008INTL, CA, USA) at 120 °C for 10 min. After blocking endogenous peroxidase and non-specific binding, sections were incubated with primary antibodies (supplementary table 5), followed by HRP-conjugated secondary antibodies (Agilent) and DAB development. Slides were counterstained with hematoxylin, dehydrated, mounted, and scanned using a Hamamatsu whole-slide scanner for image analysis with NDP.view2 Plus (v2.9.25).

### Antibody panel design and conjugation

For IMC staining, a panel of 38 metal-conjugated antibodies was applied to detect diverse cellular identities and functional states in tissue sections. Antibody clones were selected based on previously published protocols and in-house validation performed in collaboration with the Diagnostic Pathology Service at Haukeland University Hospital. Conjugation of antibodies to lanthanide metal isotopes was carried out in-house using the manufacturer’s protocol (Standard Biotools, CA, USA)^40^.

The antibody panel, detailed in Supplementary Table 6, includes markers for fibroblasts, immune cells, epithelial cells, and endothelial cells, along with functional markers related to apoptosis, proliferation, and inflammation. Additionally, commercially pre-conjugated antibodies were incorporated to facilitate downstream single-cell segmentation.

### IMC data acquisition and preprocessing

Raw imaging data were acquired using the Hyperion Imaging System (Standard BioTools Inc., South San Francisco, CA, USA) in combination with CyTOF software v7.0, following the manufacturer’s standard protocol. Image quality and marker expression were initially evaluated using MCD Viewer (supplementary Fig. 6 and Fig. 1). Raw data files (txt) were converted into OME-TIFF format using the IMC Converter tool for downstream analysis.

The resulting multi-channel images were imported into QuPath for preprocessing. This included tissue-level annotation, region definition, single-cell segmentation, marker positivity training, and preliminary spatial proximity assessment.

Data transformation and censoring were performed in RStudio. Per-cell marker intensities were winsorized per marker at the 0.1th and 99.5th percentiles to reduce the influence of extreme outliers, followed by an arcsinh transformation with a cofactor of 5.

### Region annotation and cell classification using QuPath

Tissue sections were manually and algorithmically segmented into three distinct regions: vessel, epithelium, and stroma. Region segmentation was performed using AI-based models in QuPath, trained on representative regions from the full dataset^41^.

Single-cell segmentation was performed using InstanSeg^42^, which utilized five input imaging channels: DNA1, DNA2, Marker1, Marker2, and Marker3. Cells were segmented separately within each region. For cell classification, QuPath’s object classifier was applied to determine marker positivity on a per-cell basis. Marker expression was evaluated using a combination of AI-driven classification and manual verification. Positivity for most markers was determined based on whole-cell signal, while YAP1 expression was assessed specifically within the nucleus. Multiple markers were used simultaneously for classifying per cell.

Spatial relationships between cells and annotated regions were quantified using the “Signed distance to annotations 2D” function in QuPath, enabling further analysis of cell infiltration patterns and local cell density. All measured data were then exported for downstream analysis.

### Supervised clustering of single cells

Supervised clustering^43^ was performed in RStudio, building upon the initial classification results generated in QuPath. Cells were classified across four hierarchical levels. In Level 1, cells were categorized into five basic types: immune cells, endothelial cells, epithelial cells, fibroblasts, and functional cells. Cells not assigned to any of these categories were labeled as unclassified, which occupied 4.2%. Immune cells were further subdivided into immune cell subtypes based on marker expressions as Level 2. To account for immune cells potentially infiltrating other tissue compartments, co-expression with endothelial, epithelial, or fibroblast markers was evaluated in Level 3. Functional states were annotated based on expression of specific markers, including apoptosis, autophagy, proliferation, migration, transcriptional activity, and pro-inflammatory function in Level 4.

To ensure data quality, extreme outlier values were excluded during preprocessing. A heatmap was generated to validate the biological plausibility of cell classification. Dimensionality reduction and visualization were performed using the scatter package in R. A UMAP projection was constructed based on marker expression^44^, with parameters set to n_neighbors = 30 and min_dist = 0.3, adjusted to suit the dataset characteristics. All scripts used for clustering and visualization are provided in the supplementary materials and Zenodo.

### Spatial analysis by SpicyR package

Spatial neighborhood analysis was conducted using the SpicyR package (v1.2.1)^15,45^ to evaluate spatial associations between different cell types in Control and ONJ tissue samples. This method models pairwise spatial co-localization patterns while accounting for inter-sample variability. Besides basic cell type, immune subtype and functional cell relationships, the spatial information between immune subtype and basic cell was also examined. Cell centroid coordinates (x, y), extracted from imaging data, served as the basis for all spatial calculations. Spatial radii (R_s_) of 50 μm and 100 μm were applied to capture both immediate and intermediate cellular interactions.

### Differential cell type and functional marker analysis

To evaluate differences in cellular composition and functional marker expression between Control and ONJ groups, a comprehensive differential analysis was performed.

For cellular composition, single-cell annotations were aggregated to obtain per-sample proportions for each cell class across all classification levels. For each cell type, the distribution of proportions in Control and ONJ groups was visualized using box-and-whisker plots. A panel of functional markers was analyzed, including PD1, YAP1, BNIP3, pNFKB, Caveolin, Ki67, pERK, IntegrinB1, and pSTAT3. Expression data were obtained from the merged dataset and mapped to the corresponding region and group.

### Statistics and reproducibility

IMC tissue sections were analyzed from 14 individuals. For each individual, 2–5 FFPE tissue cores or regions of interest (ROIs) were acquired and processed. IHC included 7 individuals, with 2–10 sections per case stained and evaluated for the selected markers. Biological replicates were defined as distinct individual, whereas technical replicates were defined as multiple ROIs.

Statistical analyses and visualizations were performed in R using standard packages for data manipulation and plotting. All quantitative analyses were conducted in a batch-processing framework, and consistent patterns across individuals supported the robustness and reproducibility of the observations. The Wilcoxon rank-sum test was used as the primary method for non-parametric group comparison. Two-sample t-test was applied for confirmation. P-values were adjusted for multiple testing using the Benjamini–Hochberg (BH) method. Significance levels are indicated in the figures as *** (*P* < 0.001), ** (*P* < 0.01), * (*P* < 0.05), and “ns.” for non-significant comparisons.

### Reporting summary

Further information on research design is available in the Nature Portfolio Reporting Summary linked to this article.

### Ethics

The study was approved by the Regional Committees for Medical and Health Research Ethics (REK, approval number 2018/716). Informed consent was obtained from all human research participants.

## Supplementary information

Supplementary Information

Description of Additional Supplementary Files

Supplementary software

Reporting summary

## Data Availability

De-identified IMC raw data and Supplementary Data have been deposited in Figshare^46^ under the DOI 10.6084/m9.figshare.30383407. Source data underlying figures are provided with this paper as Supplementary Materials.

## Code availability

Code used for cell classification, clustering, spatial analysis, and statistical analysis in this study is provided as Supplementary Code files and is archived in Zenodo^47^ (DOI: 10.5281/zenodo.18290925). The Zenodo record contains the exact version of the code used to generate the reported results. Analyses were performed in R (RStudio 4.4.1).

## References

[1] Seo, D. D. & Borke, J. L. Medication-related osteonecrosis of the jaw – 2024 update. *Oral Health Dent. Sci.* **8**, 10.33425/2639-9490.1142 (2024).

[2] Ruggiero, S. L. et al. American Association of Oral and Maxillofacial Surgeons’ position paper on medication-related osteonecrosis of the jaws-2022 update. *J. Oral. Maxillofac. Surg.* **80**, 920–943 (2022). 35300956 10.1016/j.joms.2022.02.008

[3] He, L., Sun, X., Liu, Z., Qiu, Y. & Niu, Y. Pathogenesis and multidisciplinary management of medication-related osteonecrosis of the jaw. *Int. J. Oral. Sci.* **12**, 30 (2020). 33087699 10.1038/s41368-020-00093-2 PMC7578793

[4] Roato, I. et al. Immune dysfunction in medication-related osteonecrosis of the jaw. *Int. J. Mol. Sci.* **24**, 10.3390/ijms24097948 (2023). 10.3390/ijms24097948 PMC10177780 37175652

[5] Allegra, A. et al. Patients with bisphosphonates-associated osteonecrosis of the jaw have reduced circulating endothelial cells. *Hematol. Oncol.* **25**, 164–169 (2007). 17577204 10.1002/hon.819

[6] Srivichit, B., Thonusin, C., Chattipakorn, N. & Chattipakorn, S. C. Impacts of bisphosphonates on the bone and its surrounding tissues: mechanistic insights into medication-related osteonecrosis of the jaw. *Arch. Toxicol.* **96**, 1227–1255 (2022). 35199244 10.1007/s00204-021-03220-y

[7] Wu, M. et al. Spatial proteomics: unveiling the multidimensional landscape of protein localization in human diseases. *Proteome Sci.* **22**, 7 (2024). 39304896 10.1186/s12953-024-00231-2 PMC11416001

[8] Nunes, J. B. et al. Integration of mass cytometry and mass spectrometry imaging for spatially resolved single-cell metabolic profiling. *Nat. Methods* **21**, 1796–1800 (2024). 39210066 10.1038/s41592-024-02392-6 PMC11466816

[9] Mund, A., Brunner, A. D. & Mann, M. Unbiased spatial proteomics with single-cell resolution in tissues. *Mol. Cell* **82**, 2335–2349 (2022). 35714588 10.1016/j.molcel.2022.05.022

[10] Chang, Q. et al. Imaging mass cytometry. *Cytom. A* **91**, 160–169 (2017). 10.1002/cyto.a.23053 28160444

[11] Baharlou, H., Canete, N. P., Cunningham, A. L., Harman, A. N. & Patrick, E. Mass cytometry imaging for the study of human diseases-applications and data analysis strategies. *Front. Immunol.* **10**, 2657 (2019). 31798587 10.3389/fimmu.2019.02657 PMC6868098

[12] Patel, S. S. & Rodig, S. J. Overview of tissue imaging methods. *Methods Mol. Biol.* **2055**, 455–465 (2020). 31502165 10.1007/978-1-4939-9773-2_21

[13] Rogenes, H. et al. Development of 42 marker panel for in-depth study of cancer associated fibroblast niches in breast cancer using imaging mass cytometry. *Front. Immunol.* **15**, 1325191 (2024). 38711512 10.3389/fimmu.2024.1325191 PMC11070582

[14] Bankhead, P. et al. QuPath: Open source software for digital pathology image analysis. *Sci. Rep.* **7**, 16878 (2017). 29203879 10.1038/s41598-017-17204-5 PMC5715110

[15] Canete, N. P. et al. spicyR: spatial analysis of in situ cytometry data in R. *Bioinformatics* **38**, 3099–3105 (2022). 35438129 10.1093/bioinformatics/btac268 PMC9326848

[16] Liu, M., Zhao, S., Lin, Q. & Wang, X. P. YAP regulates the expression of Hoxa1 and Hoxc13 in mouse and human oral and skin epithelial tissues. *Mol. Cell Biol.* **35**, 1449–1461 (2015). 25691658 10.1128/MCB.00765-14 PMC4372702

[17] Cerrato, A. et al. Actinomyces and MRONJ: a retrospective study and a literature review. *J. Stomatol. Oral. Maxillofac. Surg.* **122**, 499–504 (2021). 32827811 10.1016/j.jormas.2020.07.012

[18] Shibahara, T. Antiresorptive agent-related osteonecrosis of the jaw (ARONJ): a twist of fate in the bone. *Tohoku J. Exp. Med.* **247**, 75–86 (2019). 30713280 10.1620/tjem.247.75

[19] Tan, C. L. et al. PD-1 restraint of regulatory T cell suppressive activity is critical for immune tolerance. *J. Exp. Med.* **218**, 10.1084/jem.20182232 (2021). 10.1084/jem.20182232 PMC7543091 33045061

[20] Ciobanu, G. A. et al. Correlations between immune response and etiopathogenic factors of medication-related osteonecrosis of the jaw in cancer patients treated with zoledronic acid. *Int. J. Mol. Sci.* **24**, 10.3390/ijms241814345 (2023). 10.3390/ijms241814345 PMC10532296 37762651

[21] Chen, R. Y. et al. The role of PD-1 signaling in health and immune-related diseases. *Front. Immunol.* **14**, 1163633 (2023). 37261359 10.3389/fimmu.2023.1163633 PMC10228652

[22] Wynn, T. A. & Vannella, K. M. Macrophages in tissue repair, regeneration, and fibrosis. *Immunity* **44**, 450–462 (2016). 26982353 10.1016/j.immuni.2016.02.015 PMC4794754

[23] Hanna, B. S., Yaghi, O. K., Langston, P. K. & Mathis, D. The potential for Treg-enhancing therapies in tissue, in particular skeletal muscle, regeneration. *Clin. Exp. Immunol.* **211**, 138–148 (2023). 35972909 10.1093/cei/uxac076 PMC10019136

[24] Zhao, W. et al. Immunosuppressive functions of M2 macrophages derived from iPSCs of patients with ALS and Healthy Controls. *iScience* **23**, 101192 (2020). 32521508 10.1016/j.isci.2020.101192 PMC7286967

[25] Wang, J. et al. Human FOXP3 and tumour microenvironment. *Immunology* **168**, 248–255 (2023). 35689826 10.1111/imm.13520

[26] Koltsida, O. et al. Toll-like receptor 7 stimulates production of specialized pro-resolving lipid mediators and promotes resolution of airway inflammation. *EMBO Mol. Med.* **5**, 762–775 (2013). 23584892 10.1002/emmm.201201891 PMC3662318

[27] Virtej, A. et al. Contribution of initial lymphatics to oral wound healing after tooth extraction. *Eur. J. Oral. Sci.* **132**, e13006 (2024). 38989803 10.1111/eos.13006

[28] Sun, X. & Kaufman, P. D. Ki-67: more than a proliferation marker. *Chromosoma* **127**, 175–186 (2018). 29322240 10.1007/s00412-018-0659-8 PMC5945335

[29] Xu, Q. X. et al. Aberrant activation of Notch1 signaling in the mouse uterine epithelium promotes hyper-proliferation by increasing estrogen sensitivity. *FASEB J.* **37**, e22983 (2023). 37249327 10.1096/fj.202201868RR PMC10263383

[30] Ishikawa, K., Chambers, J. K. & Uchida, K. Activation of the Wnt/β-catenin signaling pathway and CTNNB1 mutations in canine intestinal epithelial proliferative lesions. *J. Vet. Med. Sci.* **86**, 748–755 (2024). 38811188 10.1292/jvms.24-0125 PMC11251820

[31] Lüönd, F. et al. Hierarchy of TGFβ/SMAD, Hippo/YAP/TAZ, and Wnt/β-catenin signaling in melanoma phenotype switching. *Life Sci. Alliance* **5**, 10.26508/lsa.202101010 (2022). 10.26508/lsa.202101010 PMC8616544 34819356

[32] Talbott, H. E., Mascharak, S., Griffin, M., Wan, D. C. & Longaker, M. T. Wound healing, fibroblast heterogeneity, and fibrosis. *Cell Stem Cell* **29**, 1161–1180 (2022). 35931028 10.1016/j.stem.2022.07.006 PMC9357250

[33] Li, Q., Ouyang, X. & Lin, J. The impact of periodontitis on vascular endothelial dysfunction. *Front. Cell Infect. Microbiol.* **12**, 998313 (2022). 36118034 10.3389/fcimb.2022.998313 PMC9480849

[34] Li, Y. et al. BNIP3L/NIX-mediated mitophagy: molecular mechanisms and implications for human disease. *Cell Death Dis.* **13**, 14 (2021). 34930907 10.1038/s41419-021-04469-y PMC8688453

[35] McQuiston, A. & Diehl, J. A. Recent insights into PERK-dependent signaling from the stressed endoplasmic reticulum. *F1000Res* **6**, 1897 (2017). 29152224 10.12688/f1000research.12138.1 PMC5664976

[36] Dalton, C. M., Schlegel, C. & Hunter, C. J. Caveolin-1: a review of intracellular functions, tissue-specific roles, and epithelial tight junction regulation. *Biology* **12**, 10.3390/biology12111402 (2023). 10.3390/biology12111402 PMC10669080 37998001

[37] Sun, L., Guo, S., Xie, Y. & Yao, Y. The characteristics and the multiple functions of integrin β1 in human cancers. *J. Transl. Med.* **21**, 787 (2023). 37932738 10.1186/s12967-023-04696-1 PMC10629185

[38] Zhang, J. & Ney, P. A. Role of BNIP3 and NIX in cell death, autophagy, and mitophagy. *Cell Death Differ.* **16**, 939–946 (2009). 19229244 10.1038/cdd.2009.16 PMC2768230

[39] Tošić, I. & Frank, D. A. STAT3 as a mediator of oncogenic cellular metabolism: pathogenic and therapeutic implications. *Neoplasia* **23**, 1167–1178 (2021). 34731785 10.1016/j.neo.2021.10.003 PMC8569436

[40] Tornaas, S. et al. Development of a high dimensional imaging mass cytometry panel to investigate spatial organization of tissue microenvironment in formalin-fixed archival clinical tissues. *Heliyon* **10**, e31191 (2024). 38803925 10.1016/j.heliyon.2024.e31191 PMC11128903

[41] Franken, A., Bila, M. & Lambrechts, D. Protocol for whole-slide image analysis of human multiplexed tumor tissues using QuPath and R. *STAR Protoc*. **5**, 10.1016/j.xpro.2024.103270 (2024). 10.1016/j.xpro.2024.103270 PMC11541771 39453815

[42] Goldsborough, T. et al. A novel channel invariant architecture for the segmentation of cells and nuclei in multiplexed images using InstanSeg. *bioRxiv*. 10.1101/2024.09.04.611150 (2024).

[43] Lee, J. T. H. & Hemberg, M. Supervised clustering for single-cell analysis. *Nat. Methods* **16**, 965–966 (2019). 31501544 10.1038/s41592-019-0534-4

[44] Becht, E. et al. Dimensionality reduction for visualizing single-cell data using UMAP. *Nat. Biotechnol.*10.1038/nbt.4314 (2018). 10.1038/nbt.4314 30531897

[45] Erreni, M. et al. From surfing to diving into the tumor microenvironment through multiparametric imaging mass cytometry. *Front. Immunol.* **16**, 10.3389/fimmu.2025.1544844 (2025). 10.3389/fimmu.2025.1544844 PMC12021836 40292277

[46] Cai, J. et al. Raw data and supplementary data of imaging mass cytometry unveils functional and spatial remodeling of peri-lesional cells in jaw osteonecrosis. [Data set]. *Figshare*. 10.6084/m9.figshare.30383407 (2026). 10.1038/s42003-026-09696-7 PMC13021915 41699257

[47] Cai, J. et al. R code for analyzing IMC data processed by QuPath. [Data set]. *Zenodo*. 10.5281/zenodo.18290925 (2026).

