# Supplementary material for: Imaging mass cytometry unveils functional and spatial remodeling of peri-lesional cells in jaw osteonecrosis
Source: Commun Biol. 2026 Feb 16;9:442. doi: 10.1038/s42003-026-09696-7 (PMC13021915; doi:10.1038/s42003-026-09696-7)
Supplement: Supplementary file 1 — Supplementary Information [file 42003_2026_9696_MOESM1_ESM.pdf]

## Supplementary Information

# Imaging Mass Cytometry Unveils Functional and Spatial Remodeling of Peri-lesional Cells in Jaw Osteonecrosis

Jiazheng Cai [1,#], Ying Xue [2,#], Stian Tornaas[3], Harsh Nitin Dongre[3], Athanasia Bletsa[4], Sigbjørn Løes[2,5], Peter Schleier[6], Evelyn Neppelberg[4,5], Arild Kvalheim[7], Ellen Berggreen[8], Daniela-Elena Costea [3,9], Zhe Xing [1,*] and Anca Virtej [2]

[1] Department of Medical Biology, Faculty of Health Sciences, UiT the Arctic University of Norway, 9037, Tromsø, Norway; Jiazheng.Cai@uit.no (J.C.); zhe.xing@uit.no (Z.X.)
[2] Department of Clinical Dentistry, Faculty of Health Sciences, UiT the Arctic University of Norway, 9037, Tromsø, Norway; ying.xue@uit.no (Y.X.); anca.virtej@uit.no (A.V.); sigbjorn.loes@uit.no (S.L.)
[3] Gade Laboratory for Pathology and Norwegian Center for Cancer Biomarkers CCBIO, Department of Clinical Medicine, Faculty of Medicine, University of Bergen, 5009, Bergen, Norway; daniela.costea@uib.no (D.C.); stiantf@gmail.com (S.T.); Harsh.Dongre@uib.no (H.N.D.)
[4] Department of Clinical Dentistry, University of Bergen, 5009, Bergen, Norway; nancy.bletsa@uib.no (A.B), evelyn.neppelberg@uib.no (E.N.)
[5] Department of Oral and Maxillofacial Surgery, Haukeland University Hospital, 5009, Bergen, Norway
[6] Department of Otolaryngology, Stavanger University Hospital, 4011, Stavanger, Norway; Schleier@online.no (P.S.)
[7] Oris Dental Tannteam Secialist Clinic, 5221, Nesttun, Norway; arild.kvalheim@orisdental.no (A.K.)
[8] Department of Biomedicine, University of Bergen, 5006, Bergen, Norway; ellen.berggreen@uib.no (E.B.)
[9] Department of Pathology, Haukeland University Hospital, 5021, Bergen, Norway

# These authors contributed equally

* Correspondence:

Zhe Xing

Email: zhe.xing@uit.no

Supplementary figure 1. Observation of Control and ONJ sample from QuPath. A. is the region's segmentation results, including stroma (Yellow), vessel (red) and epithelial (blue). B.is the image after cell segmentation and annotation. different color means different cell type. C. is each cell's distance to vessel regions. The threshold is from -20 to 20. White cell means away from vessel, the greener of the cells, the closer to vessel region. Scale bar: 100 μm.

Supplementary figure 2. Heatmap marker expression of 4-layer cell types.

Supplementary figure 3. Nuclei and collagen expression in Control and ONJ groups.

Supplementary figure 4. UMAP by individual sample ID and selected marker expression.

Supplementary figure 5. Core tissue micro-array (cTMA) of IMC. The blue frame represents Tonsil, the red frame represents ONJ patients, and the green frame represents control samples.

Supplementary figure 6. Simultaneous visualization of various immune cell subsets in a tonsil core from cTMA: CD8+T cell (red, CD8+), CD4+T cell (green, CD4+), macrophages (magenta, CD68+), B cell (cyan, CD20+), and regulatory T-cells (blue, CD4+, FoxP3+). Scale bar: 100 μm.

Supplementary Table 1. Statistical analysis of cell type percentage in three regions: Control vs ONJ in basic and function groups.

Supplementary Table 2. Functional markers express: Control vs ONJ in three basic cell types.

Supplementary Table 3. cell classification logic and the number of each cell type. Total number and proportion include Control, ONJ and Tonsil group.

Supplementary Table 4. Sample Overview: detailed information of different Control and ONJ samples.

Supplementary Table 5. Antibodies of IHC staining.

Supplementary Table 6. Overview of IMC markers included in the study. α-SMA: alpha-smooth muscle actin, EGFR: Epithelial growth factor receptor, YAP1: yes-associated protein-1, FAP: fibroblast activation protein, pERK: Protein kinase-like endoplasmic reticulum kinase, FSP-1: fibroblast specific protein.

Supplementary Table 7. SpicyR output table for pairwise cell-type spatial associations

Supplementary Table 8. Cell type differential analysis results in four levels.

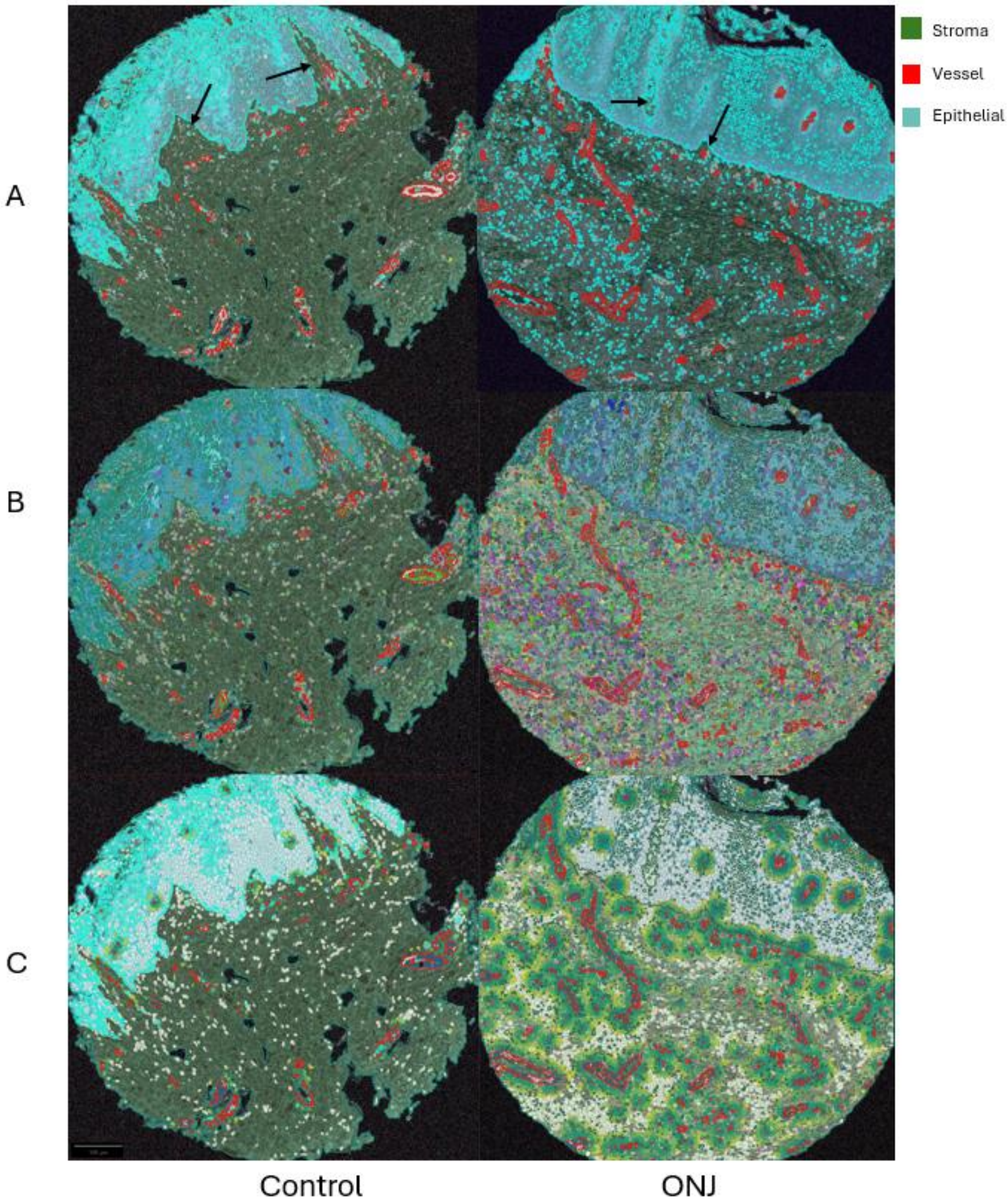


Supplementary figure 1. Observation of Control and ONJ sample from QuPath. A. is the region's segmentation results, including stroma (Yellow), vessel (red) and epithelial (blue). Black arrows: interdigitated junction between epithelium and connective tissue. B.is the image after cell segmentation and annotation. Different color means different cell type. C. is each cell's distance to vessel regions.  The threshold is from -20 to 20. White cell means away from vessel, the greener of the cells, the closer to vessel region. Scale bar: 100 μm.

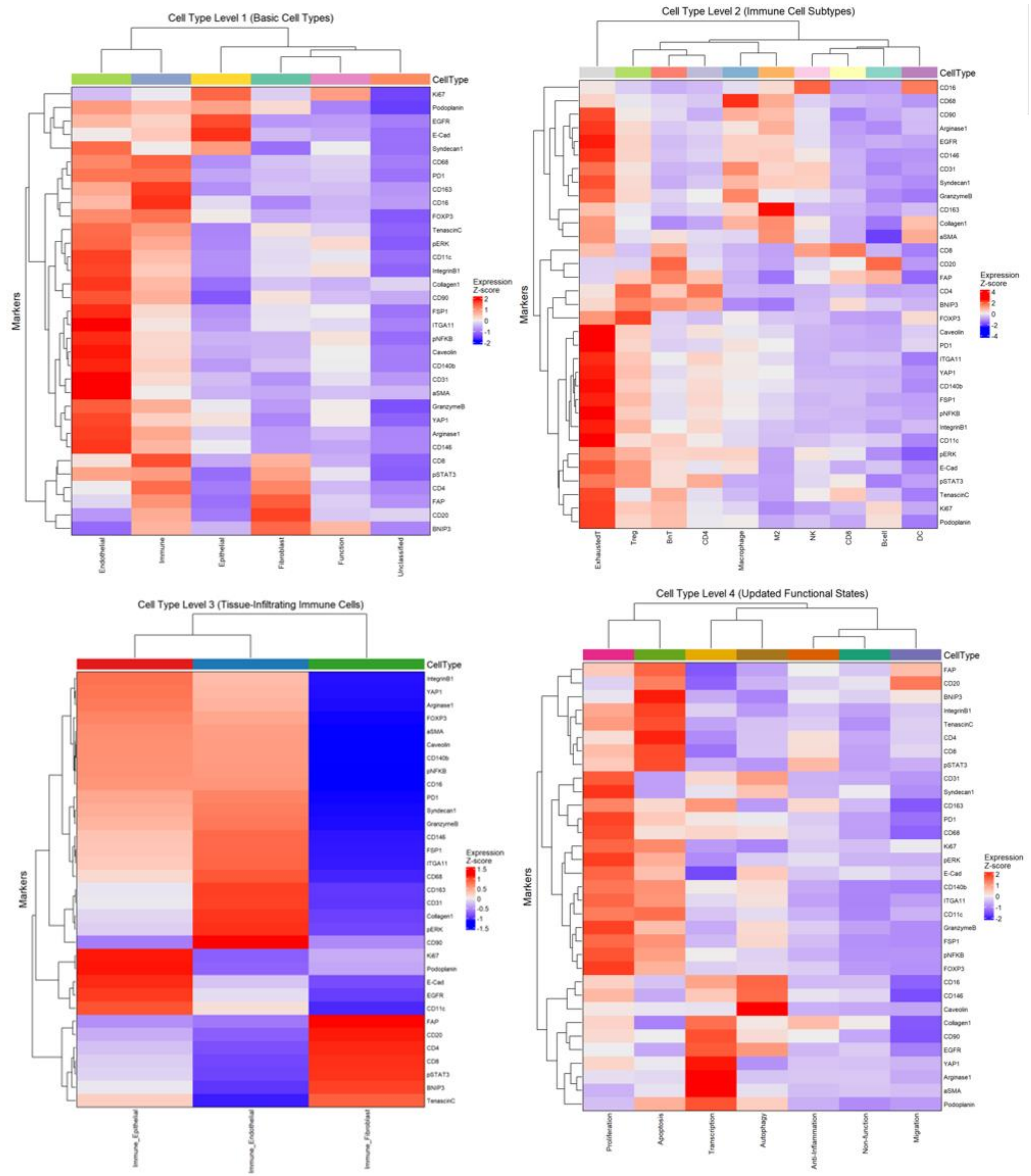


Supplementary figure 2. Heatmap marker expression of 4-layer cell types.

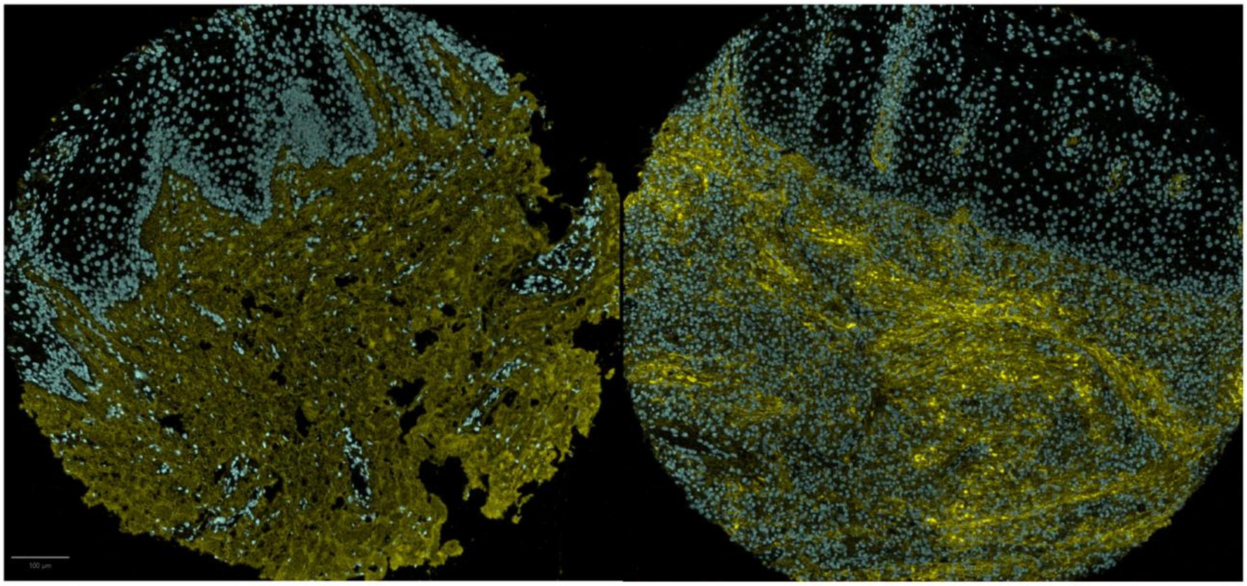

Control ONJ

Supplementary figure 3. Nuclei and collagen expression in Control and ONJ groups. Nuclei are shown in blue and collagen in yellow. In Control, nuclei are sparsely distributed, and collagen is uniformly present throughout the stromal compartment. In ONJ tissues, nuclei exhibit intensive, while collagen appears unevenly distributed. Scale bar: 100 µm.

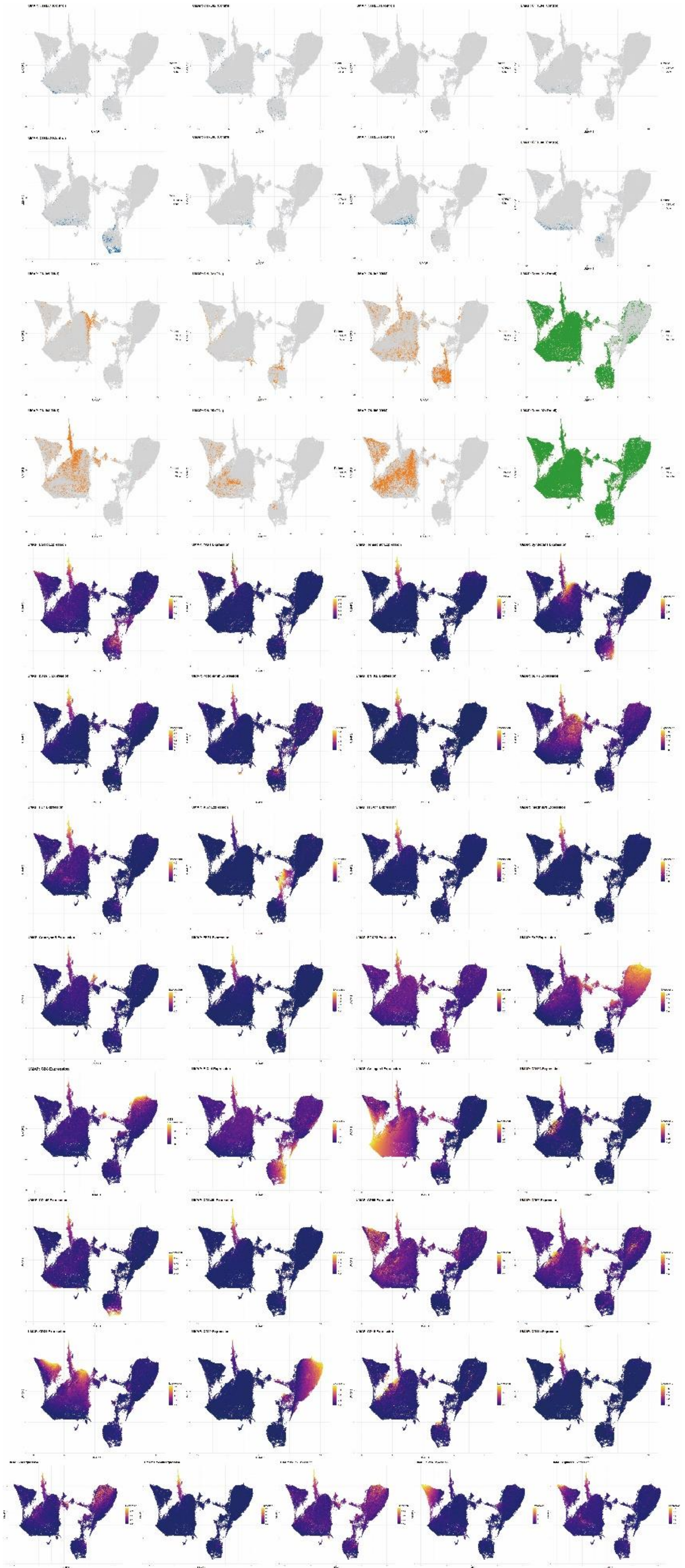

Supplementary figure 4. UMAP by individual sample ID and selected marker expression.

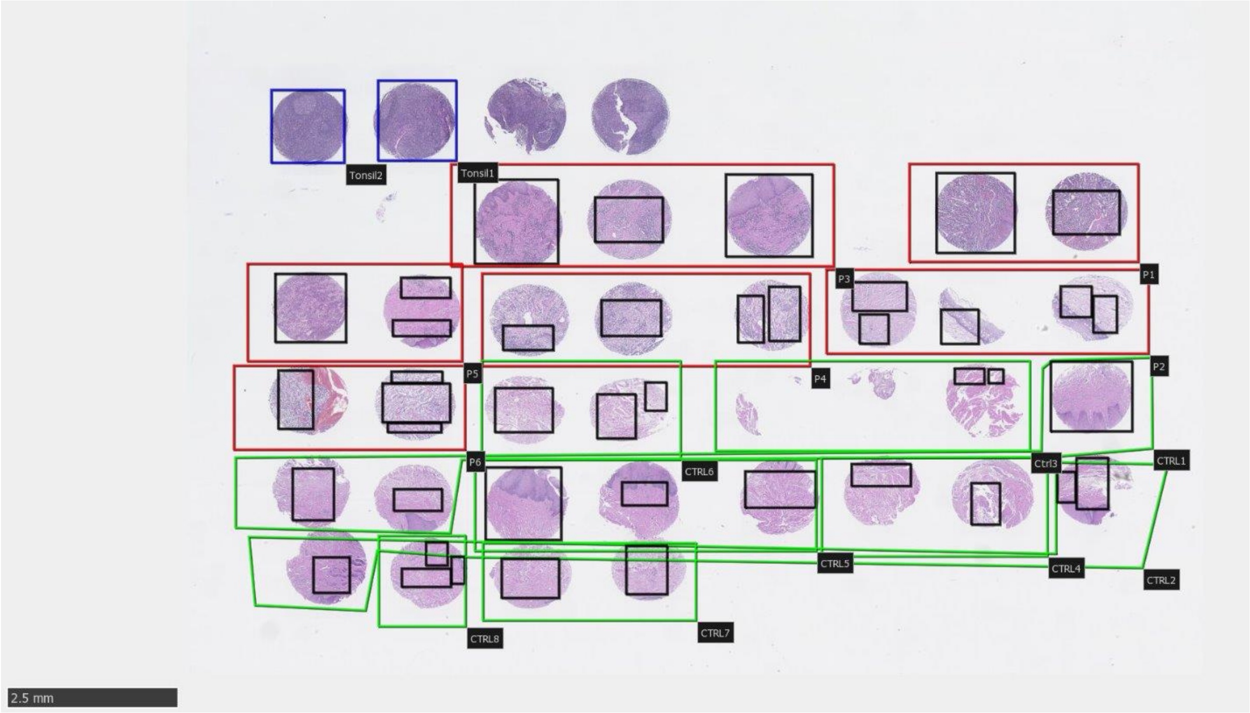


Supplementary figure 5. Core tissue micro-array (cTMA) of IMC. The blue frame represents Tonsil, the red frame represents ONJ patients, and the green frame represents control samples.

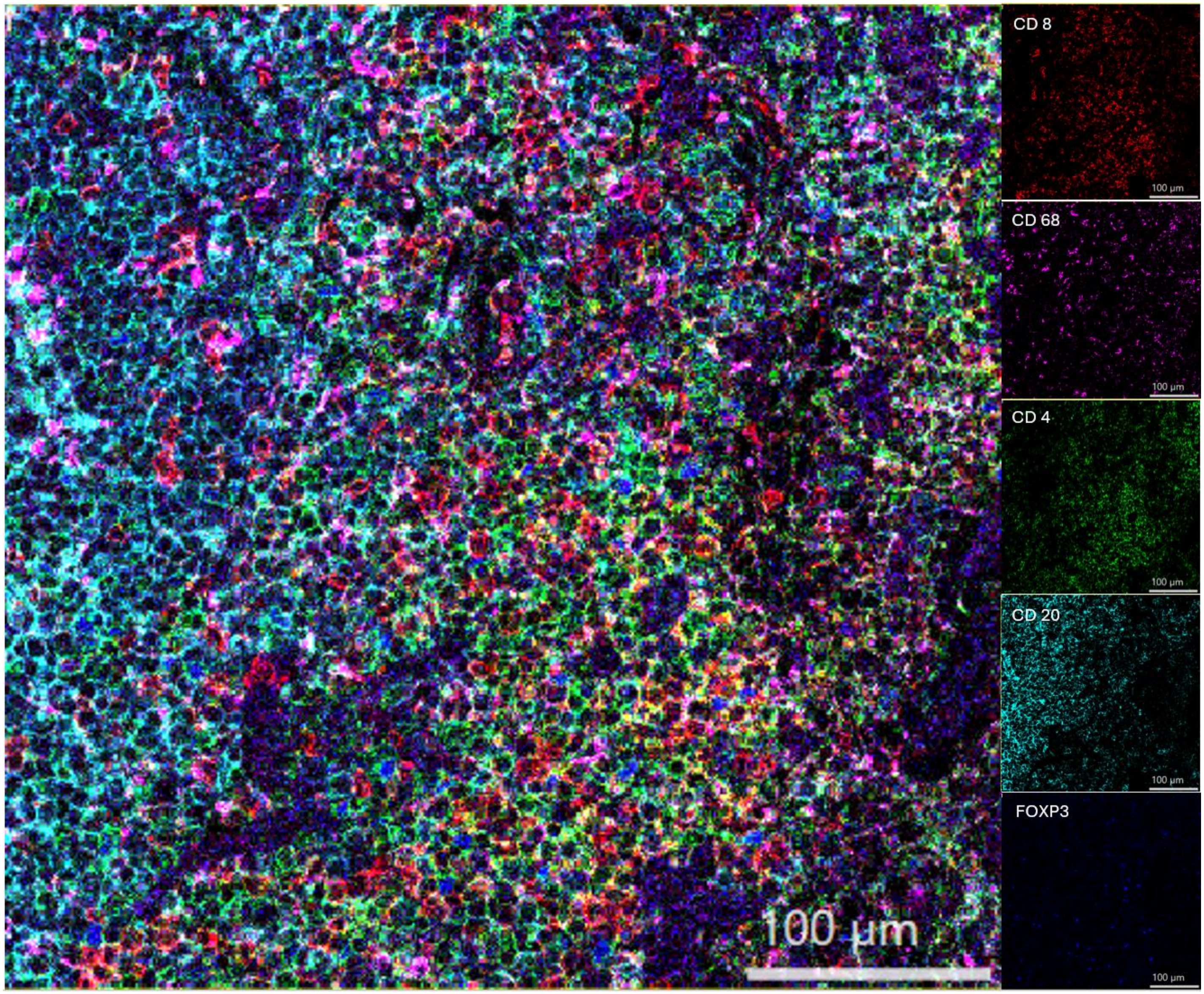


Supplementary figure 6. Simultaneous visualization of various immune cell subsets in a tonsil core from cTMA: CD8+T cell (red, CD8+), CD4+T cell (green, CD4+), macrophages (magenta, CD68+), B cell (cyan, CD20+), and regulatory T-cells (blue, CD4+, FoxP3+). Scale bar: 100 µm.

Supplementary Table 1. Statistical analysis of cell type percentage in three regions: Control vs ONJ in basic and function groups.

| Level_name | Annotation | Celltype | Fisher_p_adjusted | Fisher_significance | Control_percentage | Onj_percentage | Effect_size_percentage |
|---|---|---|---|---|---|---|---|
| Basic | Epithelial | Epithelial | 0 | *** | 65.7 | 19.3 | -46.4 |
| Basic | Epithelial | Immune | 0 | *** | 10.8 | 40 | 29.2 |
| Basic | Epithelial | Endothelial | 3.9643E-94 | *** | 4.2 | 12 | 7.8 |
| Basic | Epithelial | Fibroblast | 1.8881E-60 | *** | 2.1 | 6.8 | 4.7 |
| Basic | Epithelial | Function | 2.7439E-55 | *** | 8 | 14.8 | 6.8 |
| Basic | Epithelial | Unclassified | 1.1537E-08 | *** | 9.1 | 7 | -2.1 |
| Basic | Stroma | Immune | 0 | *** | 21.5 | 42 | 20.5 |
| Basic | Stroma | Unclassified | 0 | *** | 36.9 | 10.7 | -26.2 |
| Basic | Stroma | Endothelial | 6.134E-146 | *** | 11.2 | 19.4 | 8.2 |
| Basic | Stroma | Epithelial | 1.619E-135 | *** | 11.4 | 5.6 | -5.8 |
| Basic | Stroma | Function | 9.0058E-20 | *** | 6 | 8.1 | 2.1 |
| Basic | Stroma | Fibroblast | 6.5875E-05 | *** | 13 | 14.2 | 1.2 |
| Basic | Vessels | Epithelial | 3.0476E-52 | *** | 11.8 | 3.7 | -8.1 |
| Basic | Vessels | Unclassified | 3.1735E-33 | *** | 12.1 | 5.2 | -6.9 |
| Basic | Vessels | Endothelial | 1.6532E-16 | *** | 33.2 | 41.7 | 8.5 |
| Basic | Vessels | Fibroblast | 1.757E-15 | *** | 3.9 | 7.9 | 4.1 |
| Basic | Vessels | Immune | 0.00050281 | *** | 33.6 | 37.2 | 3.6 |
| Basic | Vessels | Function | 0.00834888 | ** | 5.4 | 4.2 | -1.2 |
| Functional | Epithelial | Non-function | 0 | *** | 62.2 | 29.5 | -32.6 |
| Functional | Epithelial | Proliferation | 0 | *** | 19.3 | 48.5 | 29.2 |

| | | | | | | | |
|---|---|---|---|---|---|---|---|
| Functional | Epithelial | Apoptosis | 2.0486E-32 | *** | 2.9 | 6.3 | 3.4 |
| Functional | Epithelial | Transcription | 3.1737E-18 | *** | 2.5 | 4.7 | 2.3 |
| Functional | Epithelial | Migration | 1.827E-15 | *** | 7.6 | 5.1 | -2.6 |
| Functional | Epithelial | Autophagy | 0.00115284 | ** | 0.5 | 0.8 | 0.4 |
| Functional | Epithelial | Pro-Inflammation | 0.69061471 | ns | 5.2 | 5 | -0.1 |
| Functional | Stroma | Non-function | 0 | *** | 75.8 | 38.1 | -37.7 |
| Functional | Stroma | Proliferation | 0 | *** | 3.6 | 47.5 | 43.9 |
| Functional | Stroma | Transcription | 7.5256E-65 | *** | 7.9 | 4.4 | -3.5 |
| Functional | Stroma | Migration | 3.4065E-22 | *** | 1.7 | 0.8 | -0.9 |
| Functional | Stroma | Pro-Inflammation | 3.7306E-15 | *** | 5.5 | 4 | -1.5 |
| Functional | Stroma | Autophagy | 0.01673746 | * | 0.2 | 0.3 | 0.1 |
| Functional | Stroma | Apoptosis | 0.0406416 | * | 5.3 | 4.9 | -0.4 |
| Functional | Vessels | Proliferation | 1.568E-183 | *** | 4.6 | 27 | 22.3 |
| Functional | Vessels | Transcription | 1.9853E-48 | *** | 23.9 | 12.3 | -11.6 |
| Functional | Vessels | Non-function | 3.9237E-25 | *** | 56.2 | 45.2 | -11 |
| Functional | Vessels | Autophagy | 5.1903E-08 | *** | 0.5 | 1.8 | 1.3 |
| Functional | Vessels | Pro-Inflammation | 0.16264595 | ns | 5.3 | 4.7 | -0.7 |
| Functional | Vessels | Apoptosis | 0.19455251 | ns | 5.5 | 4.9 | -0.6 |
| Functional | Vessels | Migration | 0.55366576 | ns | 4 | 4.3 | 0.3 |

Supplementary Table 2. Functional markers express: Control vs ONJ in three basic cell types.

| Marker | Celltype | P_value | P_adjusted | Significance |
|---|---|---|---|---|

| | | | | |
|---|---|---|---|---|
| BNIP3 | Epithelial | 6.31E-20 | 7.74E-20 | *** |
| BNIP3 | Endothelial | 1.12E-122 | 3.36E-122 | *** |
| BNIP3 | Fibroblast | 9.54E-54 | 1.61E-53 | *** |
| Caveolin | Epithelial | 0.058870708 | 0.058870708 | . |
| Caveolin | Endothelial | 5.52E-09 | 5.97E-09 | *** |
| Caveolin | Fibroblast | 1.11E-09 | 1.25E-09 | *** |
| IntegrinB1 | Epithelial | 3.54E-54 | 6.37E-54 | *** |
| IntegrinB1 | Endothelial | 4.78E-118 | 1.29E-117 | *** |
| IntegrinB1 | Fibroblast | 2.98E-35 | 4.02E-35 | *** |
| Ki67 | Epithelial | 1.79E-65 | 3.46E-65 | *** |
| Ki67 | Endothelial | 0 | 0 | *** |
| Ki67 | Fibroblast | 1.89E-244 | 6.37E-244 | *** |
| PD1 | Epithelial | 0 | 0 | *** |
| PD1 | Endothelial | 0 | 0 | *** |
| PD1 | Fibroblast | 0 | 0 | *** |
| pERK | Epithelial | 0 | 0 | *** |
| pERK | Endothelial | 0 | 0 | *** |
| pERK | Fibroblast | 0 | 0 | *** |
| pNFKB | Epithelial | 2.28E-48 | 3.43E-48 | *** |
| pNFKB | Endothelial | 1.42E-88 | 2.95E-88 | *** |
| pNFKB | Fibroblast | 1.32E-52 | 2.10E-52 | *** |
| pSTAT3 | Epithelial | 4.33E-106 | 1.06E-105 | *** |
| pSTAT3 | Endothelial | 6.88E-48 | 9.77E-48 | *** |
| pSTAT3 | Fibroblast | 1.79E-17 | 2.10E-17 | *** |
| YAP1 | Epithelial | 7.40E-04 | 7.69E-04 | *** |
| YAP1 | Endothelial | 2.44E-27 | 3.13E-27 | *** |
| YAP1 | Fibroblast | 8.95E-94 | 2.01E-93 | *** |

Supplementary Table 3. cell classification logic and the number of each cell type. Total number and proportion include Control, ONJ and Tonsil group.

| | **Cell type** | **Marker Inclusion** | **Marker Exclusion** | **Control Number and Proportion** | **ONJ Number and Proportion** | **Total Number and Proportion** |
|---|---|---|---|---|---|---|
| Level 1<br><br>**Basic Cell Type** | Immune | CD20, CD4, CD8, Arginase1, CD163, CD68, PD1, FOXP3, CD16, GranzymeB, CD11c, Syndecan1 | FAP, FSP1, CD90, ITGA11, TenascinC, CD31, aSMA | 5587, 20.18% | 35578, 40.87% | 59151, 35.90% |
| | Epithelial | EGFR, E-Cad | FAP, FSP1, CD90, ITGA11, TenascinC | 6884, 24.86% | 8686, 9.98% | 17911, 10.87% |
| | Endothelial | CD31, CD140b, Podoplanin, CD146, aSMA | FAP, FSP1 | 3290, 11.88% | 16618, 19.09% | 21406, 13.00% |
| | Fibroblast | FAP, FSP1, CD90, ITGA11, Collagen1, TenascinC | | 2579, 9.32% | 9683, 11.12% | 28692, 17.41% |

| | | | | | | |
|---|---|---|---|---|---|---|
| | Function | BNIP3, YAP1, Ki67, pERK, pSTAT3, pNFKB, Caveolin, IntegrinB1 | | 1782, 6.44% | 8677, 9.97% | 14729, 8.93% |
| | Unclassified | Cells do not express any above markers | | 7564, 27.32% | 7803, 8.96% | 22890,13.89 % |
| Level 2 **Immune Cell Subtype Classification** **(derive from immune basic cell type)** | Bcell | CD20 | CD4, CD8 | 0 | 191 | 6772 |
| | BnT | CD20 AND (CD4 OR CD8) | | 2 | 31 | 1185 |
| | M2 | CD163 OR Arginase1 | | 3071 | 5523 | 9033 |
| | Plasmacyte | Syndecan1 | | 0 | 0 | 0 |
| | Macrophage | CD68 | | 504 | 2599 | 3852 |
| | Treg | **FOXP3** AND CD4 | CD8 | 6 | 499 | 905 |
| | ExhaustedT | **PD1** AND (CD4 OR CD8) | | 14 | 781 | 924 |
| | NK | (CD16 OR GranzymeB) AND CD8 | CD20, CD4 | 13 | 109 | 243 |
| | CD4 | CD4 | CD8 | 126 | 601 | 3462 |
| | CD8 | CD8 | CD4 | 156 | 648 | 3713 |
| | DC | CD11c | | 1 | 2 | 3 |
| Level 3 **Tissue-Infiltrating Immune Cells** **(derive from immune basic cell type)** | Immune_Epithelial | E-Cad, EGFR | FAP, FSP1, CD90, CD31 | 1452 | 10235 | 13763 |
| | Immune_Endothelial | CD31, CD146 | FAP, FSP1, CD90 | 998 | 11499 | 12984 |
| | Immune_Fibroblast | FAP, FSP1, CD90, ITGA11, Collagen1, TenascinC | | 1379 | 7634 | 23298 |
| Level 4 **Functional States** | Apoptosis | BNIP3 | YAP1, pNFKB, Caveolin | 1300 | 4649 | 7831 |
| | Proliferation | Ki67, pERK | | 2108 | 39905 | 55789 |
| | Migration | IntegrinB1 | | 948 | 2238 | 7024 |
| | Pro - Inflammation | pSTAT3, pNFKB | | 1488 | 3848 | 7831 |
| | Autophagy | Caveolin | | 78 | 535 | 643 |
| | Transcription | YAP1 | | 2304 | 4608 | 7341 |
| | Non-function | Cells do not express any functional markers | | 19460 | 31262 | 72499 |

Supplementary Table 4. Sample Overview: detailed information on different Control and ONJ samples.

| **Sample ID** | **Group** | **Remarks** |
|---|---|---|
| Ctrl 1 | Control | Parakeratinized epithelium; fibrous connective tissue |
| Ctrl 2 | Control | Similar to Ctrl 1, but more dilated vessels |
| Ctrl 3 | Control | Non-/partly parakeratinized epithelium; loose CT and muscle |
| Ctrl 4 | Control | Mixed keratinization; fibrous CT; dilated vessels |
| Ctrl 5 | Control | Mainly ortho/parakeratinized epithelium; fibrous CT |

| | | |
|---|---|---|
| Ctrl 6 | Control | Non-/partly parakeratinized; lose CT and muscle |
| Ctrl 7 | Control | Parakeratinized epithelium; fibrous CT |
| Ctrl 8 | Control | Parakeratinized epithelium; fibrous CT |
| P1 | ONJ | Mucosa near 47 |
| P2 | ONJ | Mucosa |
| P3 | ONJ | Mucosa near 47 |
| P4 | ONJ | Mucosa buccal 24 |
| P5 | ONJ | Gingiva from lower jaw |
| P6 | ONJ | Mucosa palatal defect |

Supplementary Table 5. Antibodies of IHC staining.

| Antibody | Supplier | Reference number | Titration |
|---|---|---|---|
| Arginase 1 | Cell Marque | 380R-16 | 1:100 |
| CD163 | Cell Marque | 163M-16 | 1:50 |
| CD68 | Agilent | GA63661-2 | 1:3000 |
| FOXP3 | BD Pharmingen | 560044 | 1:20 |
| CD4 | Cell Marque | 104R-16 | 1:100 |
| CD56 | Cell Marque | 156R-96 | 1:25 |
| PD1 | Cell Marque | 315M-96 | 1:100 |

Supplementary Table 6. Overview of IMC markers included in the study. α-SMA: alpha-smooth muscle actin, EGFR: Epithelial growth factor receptor, YAP1: yes-associated protein-1, FAP: fibroblast activation protein, pERK: Protein kinase-like endoplasmic reticulum kinase, FSP-1: fibroblast specific protein

| Marker | Supplier | Clone | Titration | Metal | In-house conjugation |
|---|---|---|---|---|---|
| **Arginase-1** | Fluidigm | D4E3M | 1:100 | 139La | No |
| **α-SMA** | Fluidigm | 1A4 | 1:400 | 141 Pr | No |
| **EGFR** | Fluidigm | D38B1 | 1:100 | 142 Nd | No |
| **Podoplanin** | CST | LpMab-12 | 1:500 | 143 Nd | Yes |
| **YAP1** | CST | D8H1X | 1:3000 | 144 Nd | Yes |
| **Caveolin** | CST | D46G3 | 1:2000 | 145 Nd | Yes |
| **CD16** | Fluidigm | EPR16784 | 1:50 | 146 Nd | No |
| **CD163** | Fluidigm | EDHu-1 | 1:600 | 147 Sm | No |
| **CD140β** | CST | 28E1 | 1:200 | 149 Sm | Yes |

| | | | | | |
|---|---|---|---|---|---|
| **Syndecan1** | Thermo Fisher | SP152 | 1:200 | 150Nd | Yes |
| **CD31** | Fluidigm | EPR3094 | 1:100 | 151 Eu | No |
| **FAP** | CST | E1V9V | 1:1000 | 152 Sm | Yes |
| **ITGA11** | UiB | Clone 24 | 1:200 | 153 Eu | Yes |
| **FoxP3** | Fluidigm | 236A/E7 | 1:50 | 155 Gd | No |
| **CD4** | Fluidigm | EPR6855 | 1:200 | 156 Gd | No |
| **E-cadherin** | Fluidigm | 24E10 | 1:100 | 158 Gd | No |
| **CD68** | Fluidigm | KP1 | 1:50 | 159 Tb | No |
| **CD20** | Fluidigm | H1 | 1:400 | 161 Dy | No |
| **CD8a** | Fluidigm | CD8/144B | 1:100 | 162 Dy | No |
| **Tenascin C** | Merck | BC-24 | 1:1000 | 163 Dy | Yes |
| **CD11c** | Fluidigm | D3V1E | 1:800 | 164Dy | No |
| **PD1** | Standard Biotools | EPR4877(2) | 1:100 | 165Ho | No |
| **pNF-kB** | Fluidigm | S529 | 1:200 | 166Er | No |
| **Granzyme B** | Fluidgm | EPR20129-217 | 1:100 | 167 Er | No |
| **Ki-67** | Fluidigm | B56 | 1:200 | 168 Er | No |
| **Collagen type 1** | Fluidigm | Polyclonal | 1:600 | 169 Tm | No |
| **pSTAT3** | Abcam | Polyclonal | 1:200 | 170Er | Yes |
| **pERK** | Fluidigm | D13.14.4E | 1:200 | 171 Yb | No |
| **FSP-1/S100A4** | CST | D9F9D | 1:2000 | 172 Yb | Yes |
| **BNIP3** | CST | D7U1T | 1:100 | 173 Yb | Yes |
| **IntegrinB1** | Abcam | EPR1040Y | 1:100 | 174Yb | Yes |
| **CD146** | Abcam | EPR3208 | 1:2000 | 175 Lu | Yes |
| **CD90/Thy-1** | CST | D3V8A | 1:1000 | 176 Yb | Yes |

Supplementary Table 7. SpicyR output table for pairwise cell-type spatial associations

| **Celltype** | **From** | **To** | **P.value** | **P.adj** | **Log10padj** | **Signed_log10padj** | **Neglog10padj** |
|---|---|---|---|---|---|---|---|
| | **Immune** | **Unclassified** | 5.23E-04 | 0.004708 | -2.32717 | -2.32717 | 2.327172 |
| | **Epithelial** | **Unclassified** | 0.001459 | 0.008754 | -2.0578 | -2.0578 | 2.057803 |
| | **Unclassified** | **Immune** | 4.85E-04 | 0.004708 | -2.32717 | -2.32717 | 2.327172 |
| | **Immune** | **Immune** | 0.010728 | 0.042914 | -1.3674 | -1.3674 | 1.367402 |
| | **Function** | **Immune** | 0.004219 | 0.018986 | -1.72158 | -1.72158 | 1.721575 |
| | **Endothelial** | **Immune** | 4.49E-04 | 0.004708 | -2.32717 | -2.32717 | 2.327172 |
| | **Immune** | **Function** | 0.003874 | 0.018986 | -1.72158 | -1.72158 | 1.721575 |
| | **Immune** | **Endothelial** | 3.11E-04 | 0.004708 | -2.32717 | -2.32717 | 2.327172 |
| **Level 1** | **Unclassified** | **Epithelial** | 0.001127 | 0.008111 | -2.09094 | -2.09094 | 2.090935 |
| | **BnT** | **CD8** | 0.005462 | 0.036866 | -1.43338 | 1.433375 | 1.433375 |
| **Level 2** | **BnT** | **CD4** | 3.38E-04 | 0.004888 | -2.31087 | -2.31087 | 2.31087 |

| | | | | | | | |
|---|---|---|---|---|---|---|---|
| | **M2** | **CD4** | 2.87E-04 | 0.004888 | -2.31087 | 2.31087 | 2.31087 |
| | **CD8** | **BnT** | 0.003647 | 0.029543 | -1.52955 | 1.529546 | 1.529546 |
| | **CD4** | **BnT** | 3.13E-04 | 0.004888 | -2.31087 | -2.31087 | 2.31087 |
| | **Macrophage** | **BnT** | 0.002202 | 0.019819 | -1.70291 | -1.70291 | 1.70291 |
| | **ExhaustedT** | **BnT** | 0.006698 | 0.041737 | -1.37948 | 1.379482 | 1.379482 |
| | **BnT** | **Macrophage** | 3.62E-04 | 0.004888 | -2.31087 | -2.31087 | 2.31087 |
| | **CD4** | **M2** | 1.95E-04 | 0.004888 | -2.31087 | 2.31087 | 2.31087 |
| | **M2** | **M2** | 6.35E-12 | 5.14E-10 | -9.28884 | 9.288838 | 9.288838 |
| | **Treg** | **M2** | 4.81E-04 | 0.00514 | -2.28904 | 2.289045 | 2.289045 |
| | **M2** | **Treg** | 5.08E-04 | 0.00514 | -2.28904 | 2.289045 | 2.289045 |
| | **BnT** | **ExhaustedT** | 0.005018 | 0.036866 | -1.43338 | 1.433375 | 1.433375 |
| | **Epithelial** | **BnT** | 0.00116 | 0.013808 | -1.85987 | -1.85987 | 1.859867 |
| | **M2** | **Endothelial** | 3.09E-05 | 0.001143 | -2.94185 | -2.94185 | 2.941849 |
| | **Treg** | **Endothelial** | 7.22E-04 | 0.010276 | -1.98819 | -1.98819 | 1.988189 |
| | **ExhaustedT** | **Endothelial** | 1.25E-05 | 5.76E-04 | -3.23971 | -3.23971 | 3.239715 |
| | **Endothelial** | **M2** | 4.45E-05 | 0.001372 | -2.86254 | -2.86254 | 2.862545 |
| | **Endothelial** | **Treg** | 5.55E-04 | 0.009327 | -2.03026 | -2.03026 | 2.03026 |
| | **BnT** | **Epithelial** | 0.003746 | 0.034175 | -1.46629 | -1.46629 | 1.466286 |
| **Level 3** | **Endothelial** | **ExhaustedT** | 6.79E-06 | 5.76E-04 | -3.23971 | -3.23971 | 3.239715 |
| | **Non-function** | **Non-function** | 0.001612 | 0.006076 | -2.21637 | -2.21637 | 2.216365 |
| | **Anti-Inflammation** | **Non-function** | 0.00268 | 0.008753 | -2.05783 | -2.05783 | 2.057826 |
| | **Migration** | **Non-function** | 4.57E-06 | 1.42E-04 | -3.84787 | -3.84787 | 3.84787 |
| | **Proliferation** | **Non-function** | 1.66E-05 | 1.42E-04 | -3.84787 | -3.84787 | 3.84787 |
| | **Apoptosis** | **Non-function** | 0.021132 | 0.041418 | -1.38281 | -1.38281 | 1.382809 |
| | **Transcription** | **Non-function** | 0.017216 | 0.036678 | -1.4356 | -1.4356 | 1.435599 |
| | **Autophagy** | **Non-function** | 0.002463 | 0.008619 | -2.06455 | -2.06455 | 2.064555 |
| | **Non-function** | **Anti-Inflammation** | 0.003465 | 0.010611 | -1.97423 | -1.97423 | 1.974233 |
| | **Anti-Inflammation** | **Anti-Inflammation** | 0.027703 | 0.048111 | -1.31776 | -1.31776 | 1.317758 |
| | **Migration** | **Anti-Inflammation** | 8.92E-04 | 0.004855 | -2.31379 | -2.31379 | 2.313791 |
| | **Proliferation** | **Anti-Inflammation** | 1.14E-05 | 1.42E-04 | -3.84787 | -3.84787 | 3.84787 |
| | **Transcription** | **Anti-Inflammation** | 0.001152 | 0.00513 | -2.28987 | -2.28987 | 2.289868 |
| | **Non-function** | **Migration** | 9.03E-06 | 1.42E-04 | -3.84787 | -3.84787 | 3.84787 |
| | **Anti-Inflammation** | **Migration** | 0.001532 | 0.006076 | -2.21637 | -2.21637 | 2.216365 |
| | **Proliferation** | **Migration** | 1.31E-04 | 8.04E-04 | -3.09457 | -3.09457 | 3.094569 |
| **Level 4** | **Non-function** | **Proliferation** | 1.59E-05 | 1.42E-04 | -3.84787 | -3.84787 | 3.84787 |

| | | | | | | | |
|---|---|---|---|---|---|---|---|
| | **Anti-Inflammation** | **Proliferation** | 1.74E-05 | 1.42E-04 | -3.84787 | -3.84787 | 3.84787 |
| | **Migration** | **Proliferation** | 5.39E-05 | 3.78E-04 | -3.42307 | -3.42307 | 3.423069 |
| | **Proliferation** | **Proliferation** | 0.020207 | 0.041256 | -1.38451 | -1.38451 | 1.38451 |
| | **Apoptosis** | **Proliferation** | 0.009199 | 0.025043 | -1.60132 | -1.60132 | 1.601319 |
| | **Transcription** | **Proliferation** | 0.016922 | 0.036678 | -1.4356 | -1.4356 | 1.435599 |
| | **Non-function** | **Apoptosis** | 0.027968 | 0.048111 | -1.31776 | -1.31776 | 1.317758 |
| | **Proliferation** | **Apoptosis** | 0.012954 | 0.031736 | -1.49844 | -1.49844 | 1.498444 |
| | **Transcription** | **Apoptosis** | 0.028474 | 0.048111 | -1.31776 | -1.31776 | 1.317758 |
| | **Non-function** | **Transcription** | 0.01506 | 0.035139 | -1.45421 | -1.45421 | 1.454207 |
| | **Anti-Inflammation** | **Transcription** | 0.001055 | 0.00513 | -2.28987 | -2.28987 | 2.289868 |
| | **Proliferation** | **Transcription** | 0.012677 | 0.031736 | -1.49844 | -1.49844 | 1.498444 |
| | **Apoptosis** | **Transcription** | 0.024094 | 0.045408 | -1.34287 | -1.34287 | 1.342871 |
| | **Non-function** | **Autophagy** | 0.004095 | 0.011803 | -1.92802 | -1.92802 | 1.928022 |

Supplementary Table 8. Cell type differential analysis results in four levels.

| **Celltype** | | **Fold_change** | **Difference** | **Effect_size** | **Wilcox_p** | **T_test_p** | **P_value** | **P_adjusted** | **Significance** |
|---|---|---|---|---|---|---|---|---|---|
| **Level1** | **Endothelial** | 0.055732 | 0.006504 | Minimal | 0.583676 | 0.828737 | 0.583676 | 0.920712 | ns |
| | **Epithelial** | -0.60185 | -0.08231 | Small | 0.024049 | 0.099412 | 0.024049 | 0.083967 | . |
| | **Fibroblast** | 0.383223 | 0.038267 | Minimal | 0.746237 | 0.144709 | 0.746237 | 0.920712 | ns |
| | **Function** | -0.38278 | -0.0324 | Minimal | 0.920712 | 0.410099 | 0.920712 | 0.920712 | ns |
| | **Immune** | 0.278233 | 0.069932 | Minimal | 0.033587 | 0.069708 | 0.033587 | 0.083967 | . |
| **Level2** | **BnT** | -0.82239 | -0.00905 | Small | NA | NA | NA | NA | Not tested |
| | **CD4** | 0.447882 | 0.016699 | Minimal | 0.196374 | 0.287884 | 0.196374 | 0.274924 | ns |
| | **CD8** | 0.071602 | 0.003219 | Minimal | 0.455805 | 0.872302 | 0.455805 | 0.455805 | ns |
| | **DC** | 1.142107 | 0.00258 | Medium | NA | NA | NA | NA | Not tested |
| | **ExhaustedT** | 2.621526 | 0.05619 | Large | 5.38E-04 | 8.93E-04 | 5.38E-04 | 0.001885 | ** |

| | | | | | | | | | |
|---|---|---|---|---|---|---|---|---|---|
| | **M2** | -0.32705 | -0.16471 | Minimal | 0.027721 | 0.010251 | 0.027721 | 0.064682 | . |
| | **Macrophage** | 0.374909 | 0.034354 | Minimal | 0.273959 | 0.42451 | 0.273959 | 0.319618 | ns |
| | **NK** | 0.480898 | 0.00298 | Minimal | 0.090496 | 0.141486 | 0.090496 | 0.158368 | ns |
| | **Treg** | 3.210947 | 0.034599 | Large | 7.59E-05 | 1.91E-05 | 7.59E-05 | 5.32E-04 | *** |
| | **Bcell** | 14.90246 | 0.030625 | Large | NA | NA | NA | NA | Not tested |
| **Level3** | **Immune_Endothelial** | 0.163358 | 0.032268 | Minimal | 0.494396 | 0.539684 | 0.494396 | 0.741594 | ns |
| | **Immune_Epithelial** | 0.039551 | 0.009658 | Minimal | 0.851309 | 0.847666 | 0.851309 | 0.851309 | ns |
| | **Immune_Fibroblast** | -0.41396 | -0.11347 | Minimal | 0.082592 | 0.064628 | 0.082592 | 0.247776 | ns |
| **Level4** | **Pro-Inflammation** | 0.109744 | 0.00434 | Minimal | 0.708269 | 0.612911 | 0.708269 | 0.881308 | ns |
| | **Apoptosis** | -0.50742 | -0.02859 | Small | 0.881308 | 0.391397 | 0.881308 | 0.881308 | ns |
| | **Autophagy** | -0.01451 | -4.81E-05 | Minimal | 0.821372 | 0.979723 | 0.821372 | 0.881308 | ns |
| | **Migration** | 0.244137 | 0.00497 | Minimal | 0.330173 | 0.351102 | 0.330173 | 0.577802 | ns |
| | **Non-function** | -0.45352 | -0.17621 | Minimal | 0.001478 | 0.001274 | 0.001478 | 0.003448 | ** |
| | **Proliferation** | 2.106645 | 0.234859 | Large | 6.26E-05 | 1.23E-04 | 6.26E-05 | 4.38E-04 | *** |
| | **Transcription** | -0.94282 | -0.04939 | Small | 5.93E-04 | 0.001566 | 5.93E-04 | 0.002077 | ** |
