# Supplementary material for: Imaging mass cytometry unveils functional and spatial remodeling of peri-lesional cells in jaw osteonecrosis
Source: Commun Biol. 2026 Feb 16;9:442. doi: 10.1038/s42003-026-09696-7 (PMC13021915; doi:10.1038/s42003-026-09696-7)
Supplement: Supplementary file 2 — Description of Additional Supplementary Files [file 42003_2026_9696_MOESM2_ESM.pdf]

Description of Additional Supplementary Files:

File name: Supplementary Software 1

Description: All R code for data preprocessing, spatial analysis and statistics.
