# Supplementary material for: Imaging mass cytometry unveils functional and spatial remodeling of peri-lesional cells in jaw osteonecrosis
Source: Commun Biol. 2026 Feb 16;9:442. doi: 10.1038/s42003-026-09696-7 (PMC13021915; doi:10.1038/s42003-026-09696-7)
Supplement: Supplementary file 4 — Reporting summary [file 42003_2026_9696_MOESM4_ESM.pdf]

nature portfolio

Corresponding author(s): Zhe Xing; Ying Xue

Last updated by author(s): Jan 20, 2026

Reporting Summary

Nature Portfolio wishes to improve the reproducibility of the work that we publish. This form provides structure for consistency and transparency in reporting. For further information on Nature Portfolio policies, see our Editorial Policies and the Editorial Policy Checklist.

Statistics

For all statistical analyses, confirm that the following items are present in the figure legend, table legend, main text, or Methods section.

n/a	Confirmed	
☐	☒	The exact sample size (*n*) for each experimental group/condition, given as a discrete number and unit of measurement
☐	☒	A statement on whether measurements were taken from distinct samples or whether the same sample was measured repeatedly
☐	☒	The statistical test(s) used AND whether they are one- or two-sided<br>*Only common tests should be described solely by name; describe more complex techniques in the Methods section.*
☐	☒	A description of all covariates tested
☐	☒	A description of any assumptions or corrections, such as tests of normality and adjustment for multiple comparisons
☐	☒	A full description of the statistical parameters including central tendency (e.g. means) or other basic estimates (e.g. regression coefficient) AND variation (e.g. standard deviation) or associated estimates of uncertainty (e.g. confidence intervals)
☐	☒	For null hypothesis testing, the test statistic (e.g. *F*, *t*, *r*) with confidence intervals, effect sizes, degrees of freedom and *P* value noted<br>*Give P values as exact values whenever suitable.*
☐	☒	For Bayesian analysis, information on the choice of priors and Markov chain Monte Carlo settings
☐	☒	For hierarchical and complex designs, identification of the appropriate level for tests and full reporting of outcomes
☐	☒	Estimates of effect sizes (e.g. Cohen's *d*, Pearson's *r*), indicating how they were calculated

Our web collection on statistics for biologists contains articles on many of the points above.

Software and code

Policy information about availability of computer code

Data collection	CyTOF software v7.0
Data analysis	SpicyR v1.2.1

For manuscripts utilizing custom algorithms or software that are central to the research but not yet described in published literature, software must be made available to editors and reviewers. We strongly encourage code deposition in a community repository (e.g. GitHub). See the Nature Portfolio guidelines for submitting code & software for further information.

Data

Policy information about availability of data

All manuscripts must include a data availability statement. This statement should provide the following information, where applicable:

- Accession codes, unique identifiers, or web links for publicly available datasets
- A description of any restrictions on data availability
- For clinical datasets or third party data, please ensure that the statement adheres to our policy

Data have been published in Figshare (DOI: 10.6084/m9.figshare.30383407).

Research involving human participants, their data, or biological material

Policy information about studies with human participants or human data. See also policy information about sex, gender (identity/presentation), and sexual orientation and race, ethnicity and racism.

Reporting on sex and gender	Information on participants' sex or gender was not collected, in accordance with the REK (Regional Committees for Medical and Health Research Ethics, Norway) approval. The study used de-identified archival human tissue samples obtained from clinical procedures without associated demographic information. Therefore, no sex- or gender-based analyses were performed.
Reporting on race, ethnicity, or other socially relevant groupings	Race or ethnicity data were not collected, consistent with REK and institutional guidelines.
Population characteristics	The study included oral mucosa, gingiva and tonsil tissues obtained from three groups:<br>(1) patients with medication-related osteonecrosis of the jaw (ONJ) undergoing clinically indicated surgical debridement<br>(2) control individuals undergoing routine oral surgery such as third molar extraction<br>(3) tonsil specimens obtained from elective tonsillectomy, which served as positive controls<br>All tissues were formalin-fixed and paraffin-embedded before analysis. Participants were adults (≥18 years). No additional demographic, clinical, or socioeconomic data were available due to ethical restrictions.
Recruitment	Samples were collected from patients undergoing the above clinical procedures at the Department of Maxillofacial and Oral Surgery, University of Bergen / Haukeland University Hospital. Recruitment was based solely on clinical availability of surplus tissue suitable for research and participant consent. No randomization or experimental intervention
Ethics oversight	Regional Committees for Medical and Health Research Ethics (REK, Norway) approval no.: 2018/716

Note that full information on the approval of the study protocol must also be provided in the manuscript.

Field-specific reporting

Please select the one below that is the best fit for your research. If you are not sure, read the appropriate sections before making your selection.

☒ Life sciences ☐ Behavioural & social sciences ☐ Ecological, evolutionary & environmental sciences

For a reference copy of the document with all sections, see nature.com/documents/nr-reporting-summary-flat.pdf

Life sciences study design

All studies must disclose on these points even when the disclosure is negative.

Sample size	Sample size was determined by ethically available clinical specimens within the REK framework. The IMC cohort comprised 6 ONJ and 8 control cases; the IHC cohort comprised 3 ONJ and 4 control cases. Each case contributed 2–10 sections. These numbers are consistent with published IMC studies and were sufficient to detect reproducible spatial and cellular trends across groups.
Data exclusions	Regions of interest (ROIs) with insufficient viable tissue were excluded.
Replication	Biological replication was achieved across independent cases (ONJ and controls), with 2–10 sections per case.
Randomization	Randomization was not applicable. Group allocation (ONJ vs control) was determined by clinical diagnosis prior to research use. Tissue processing and imaging were performed in balanced batches.
Blinding	Investigators were not blinded to group allocation, as knowledge of diagnosis (ONJ vs control) was necessary to implement QC, region inclusion criteria,and analysis steps.<br>Bias was mitigated by: (1) scripted pipelines in QuPath/R; (2) outcome-independent, pre-specified exclusion rules; (3) secondary review of key outputs.

Reporting for specific materials, systems and methods

We require information from authors about some types of materials, experimental systems and methods used in many studies. Here, indicate whether each material, system or method listed is relevant to your study. If you are not sure if a list item applies to your research, read the appropriate section before selecting a response.

Materials & experimental systems

n/a	Involved in the study
☐	☒ Antibodies
☒	☐ Eukaryotic cell lines
☒	☐ Palaeontology and archaeology
☒	☐ Animals and other organisms
☒	☐ Clinical data
☒	☐ Dual use research of concern
☒	☐ Plants

Methods

n/a	Involved in the study
☒	☐ ChIP-seq
☒	☐ Flow cytometry
☒	☐ MRI-based neuroimaging

Antibodies

Antibodies used

A panel of 38 metal-conjugated antibodies was applied to detect diverse cellular identities and functional states in FFPE tissue sections. Of the 38 antibodies, two were nuclear markers for DNA staining and three were pre-validated control markers provided by the Hyperion Imaging System.
Other 33 antibodies were purchased as pre-conjugated reagents or were conjugated in-house following the manufacturer's protocol. A complete list of antibodies, suppliers, and metal tags is provided in Supplementary Table 6.

Validation

1. Tonsil samples were served as positive control.
2. Published literature confirmation, referencing prior IMC or IHC applications using the same clones.
3. In-house validation through parallel IHC staining and morphological assessment by a certified pathologist at Haukeland University Hospital to confirm specificity and signal localization.
4. Pre-validated antibodies supplied by the Hyperion Imaging System were used as positive technical controls.

Plants

Seed stocks

Report on the source of all seed stocks or other plant material used. If applicable, state the seed stock centre and catalogue number. If plant specimens were collected from the field, describe the collection location, date and sampling procedures.

Novel plant genotypes

Describe the methods by which all novel plant genotypes were produced. This includes those generated by transgenic approaches, gene editing, chemical/radiation-based mutagenesis and hybridization. For transgenic lines, describe the transformation method, the number of independent lines analyzed and the generation upon which experiments were performed. For gene-edited lines, describe the editor used, the endogenous sequence targeted for editing, the targeting guide RNA sequence (if applicable) and how the editor was applied.

Authentication

Describe any authentication procedures for each seed stock used or novel genotype generated. Describe any experiments used to assess the effect of a mutation and, where applicable, how potential secondary effects (e.g. second site T-DNA insertions, mosiacism, off-target gene editing) were examined.
